# Sec3 promotes the initial binary t-SNARE complex assembly and membrane fusion

**DOI:** 10.1038/ncomms14236

**Published:** 2017-01-23

**Authors:** Peng Yue, Yubo Zhang, Kunrong Mei, Shaoxiao Wang, Johannes Lesigang, Yueyao Zhu, Gang Dong, Wei Guo

**Affiliations:** 1 Department of Biology, University of Pennsylvania, Philadelphia, Pennsylvania 19104, USA; 2 Department of Medical Biochemistry, Max F. Perutz Laboratories, Medical University of Vienna, 1030 Vienna, Austria

## Abstract

The soluble *N*-ethylmaleimide-sensitive factor-attachment protein receptors (SNAREs) constitute the core machinery for membrane fusion during eukaryotic cell vesicular trafficking. However, how the assembly of the SNARE complex is initiated is unknown. Here we report that Sec3, a component of the exocyst complex that mediates vesicle tethering during exocytosis, directly interacts with the t-SNARE protein Sso2. This interaction promotes the formation of an Sso2-Sec9 ‘binary' t-SNARE complex, the early rate-limiting step in SNARE complex assembly, and stimulates membrane fusion. The crystal structure of the Sec3-Sso2 complex suggests that Sec3 binding induces conformational changes of Sso2 that are crucial for the relief of its auto-inhibition. Interestingly, specific disruption of the Sec3–Sso2 interaction in cells blocks exocytosis without affecting the function of Sec3 in vesicle tethering. Our study reveals an activation mechanism for SNARE complex assembly, and uncovers a role of the exocyst in promoting membrane fusion in addition to vesicle tethering.

## Abstract (web summary)

Membrane fusion during exocytosis is mediated by interaction between SNARE proteins on vesicles and the cell membrane, but how SNARE complex assembly is initiated remains unknown. Here, the authors show that interaction of Sec3 with Sso2 on the plasma membrane promotes formation of an Sso2–Sec9 complex, an early step in SNARE assembly, likely by inhibiting Sso2 auto-inhibition.

Intracellular membrane fusion in eukaryotic cells is mediated by the soluble *N*-ethylmaleimide-sensitive factor-attachment protein receptor (SNARE) proteins^1^. The SNARE proteins reside on the targeting membrane (t-SNAREs) and vesicles (v-SNAREs), respectively. They interact with each other to form a complex of four-helix bundle through their membrane-proximal ‘SNARE motifs'^2^. The SNARE complex brings the vesicle and the target membrane to close apposition for fusion.

For exocytosis, the t-SNAREs include two sets of proteins, syntaxins and SNAP-23 homologues. Most syntaxin proteins contain an N-terminal region that autonomously folds into a three-helix bundle (the ‘Habc domain') that interacts intramolecularly with their SNARE motif to generate a ‘closed' conformation, which, in many cases, blocks its assembly into the SNARE complex^3^,^4^,^5^,^6^,^7^,^8^,^9^,^10^. One of the best-documented examples is the yeast exocytic SNAREs, which include Sso1/2 (syntaxin homologues), Sec9 (SNAP-23 homologue) and Snc1/2 (the v-SNARE)^4^,^6^,^8^. Previous studies demonstrated that their assembly into a full complex consists of two steps: (1) binary interaction between the two t-SNAREs, Sso and Sec9; (2) the ternary interaction between the Sso/Sec9 t-SNARE complex and the v-SNARE protein Snc^4^,^8^. The rate-limiting step is the interaction between Sso and Sec9, which is hindered by the auto-inhibition of Sso. Removal of the Habc domain of Sso or mutating the linker region between Habc and the SNARE motif significantly accelerates SNARE assembly^4^,^8^. Previous studies also suggest that a similar auto-inhibition exists in mammalian and worm syntaxins, and it has been speculated that an activation mechanism exists to relieve their auto-inhibition^3^,^5^,^7^,^11^.

Elucidating the molecular mechanisms that control SNARE assembly is fundamental to our understanding of exocytosis. Over the years, regulators of membrane fusion have been intensively studied in the exocytosis field. However, most of these studies investigate fusion at a stage when the binary t-SNARE complexes are already assembled, a situation that is adapted for fast modes of secretion. The mechanisms that control the initial assembly of the binary t-SNARE complex, which applies to membrane fusion in most eukaryotic cells, remain elusive.

While the SNAREs drive fusion, the initial contact between the vesicles and their target membranes is mediated by proteins called ‘tethers'^12^,^13^,^14^. The exocyst is an evolutionarily conserved octameric protein complex that mediates the tethering of post-Golgi secretory vesicles to the plasma membrane^15^,^16^,^17^. The exocyst consists of Sec3, Sec5, Sec6, Sec8, Sec10, Sec15, Exo70 and Exo84. Work in budding yeast showed that mutations in the exocyst led to an accumulation of secretory vesicles and a block in secretion^18^. The exocyst is specifically localized to the growing end of daughter cell (the ‘bud tip') or mother-daughter cell junction (the ‘bud neck'), site of active exocytosis and surface expansion^19^. This pattern of localization stands in contrast to that of the t-SNARE proteins Sso1/2 and Sec9, which are distributed throughout the plasma membrane^20^. It is thought that the exocyst tethers the vesicles at specific regions of the plasma membrane before SNARE-mediated fusion.

Here we report that the exocyst subunit Sec3 directly interacts with Sso1/2, and promotes the initial assembly of the Sso-Sec9 t-SNARE complex and stimulates membrane fusion. We have obtained the crystal structure of the Sec3–Sso2 complex under two different crystallization conditions, both of which demonstrate that the binding of Sec3 leads to conformational changes on Sso that is crucial for the relief of its auto-inhibition. In cells, specific disruption of the Sec3–Sso interaction blocks exocytosis without affecting vesicle tethering. Our study reveals a novel mechanism for the early rate-limiting step of SNARE complex assembly and membrane fusion. Furthermore, it identifies a new role for the exocyst during exocytosis that is different from vesicle tethering.

## Results

### Sec3 binds to the t-SNARE protein Sso2

**Figure 1:**
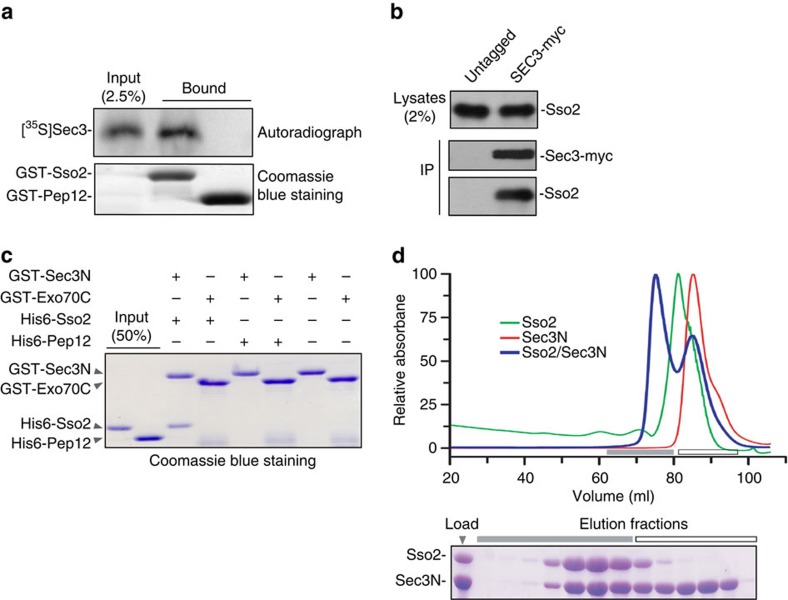
Sec3 interacts with Sso2. (**a**) Full-length Sec3 was synthesized by *in vitro* translation in the presence of [^35^S] methionine in rabbit reticulate lysates, and was used in a binding reaction with GST-tagged cytosolic domains of Sso2 or Pep12. In all, 2.5% of the total input and the bound Sec3 were analysed by SDS–PAGE and autoradiography. (**b**) Cell lysates were prepared from a yeast strain with myc-tagged Sec3 expressed under the endogenous *SEC3* promoter. The untagged strain was used as a control. The lysates were incubated with anti-myc antibodies. Sso2 co-immunoprecipitated with Sec3-myc was detected by the anti-Sso2 antibody. (**c**) His6-tagged cytosolic domains of Sso2 or Pep12 was incubated with GST-Sec3N or GST-Exo70C conjugated to glutathione Sepharose 4B. The bound proteins were analysed by SDS–PAGE and detected by Coomassie blue staining. (**d**) Size-exclusion chromatography elution profiles of Sso2 (a.a. 1–270), Sec3(a.a. 75–320), and a mixture of Sso2 with excess Sec3N. An SDS–PAGE gel showing proteins in the mixture (‘Load') and subsequent SEC peak fractions is shown in the lower panel. Lanes marked with a grey bar are fractions containing the stably formed complex. Excess Sec3N (white bar) was eluted in a later peak corresponding to unbound Sec3N.

Using the yeast 2-hybrid assay, we performed a pair-wise testing of all eight yeast exocyst subunits with the yeast t-SNARE protein Sso2. A positive interaction was found between Sec3 and Sso2, and the binding site was mapped to amino acid 1-320 of Sec3 (‘Sec3N') (Supplementary Fig. 1a). Biochemical binding assay shows that *in vitro* translated Sec3 binds to Sso2 but not the endosomal SNARE protein Pep12 (Fig. 1a). Sso2 also co-immunoprecipitated with Sec3 from cell lysates, suggesting their interaction in cells (Fig. 1b). Recombinant Sec3N bound to both Sso2 (Fig. 1c) and Sso1, a close paralog of Sso2 in yeast (Supplementary Fig. 1b). As a negative control, glutathione *S*-transferase (GST)-tagged Exo70 fragment (a.a. 358–623), which is of similar molecular weight to GST-Sec3N, did not bind to Sso2 (Fig. 1c). We have also examined the Sec3N–Sso2 interaction using size exclusion chromatography (SEC). The two purified proteins were applied to a Superdex-200 16/60 column either individually or in mixture. As shown in Fig. 1d, Sec3N and Sso2 formed a stable complex, which was later used for crystallization (see below).

### Sec3 promotes the interaction between Sso2 and Sec9

**Figure 2:**
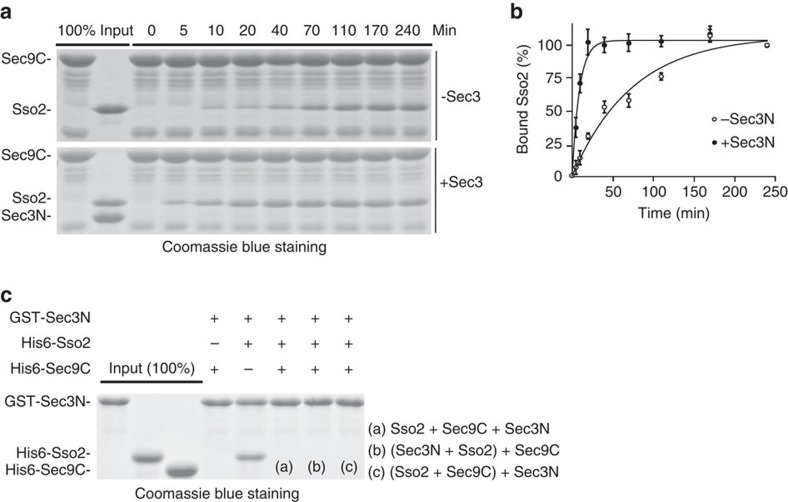
Sec3 promotes the Sso2–Sec9 interaction. (**a**) His6-Sso2, either alone (upper panel) or pre-incubated with Sec3N (lower panel), was incubated with GST-tagged C-terminal SNARE domain of Sec9 (‘GST-Sec9C') conjugated to glutathione Sepharose 4B at 25 °C. Samples were taken at indicated time points and analysed by SDS–PAGE and Coomassie blue staining. (**b**) The bound Sso2 at different time points was quantified, with bound Sso2 at 240 min without Sec3N normalized as 100%. The percentage of binding in the presence (‘+Sec3N') or absence (‘-Sec3N') of Sec3N was plotted. The points in the graph represent the average of three experiments. Error bars: s.e. (**c**) Sec3N does not form a ternary complex with Sso2 and Sec9C. GST-Sec3N was incubated with His6-Sso2 or His6-Sec9C for binding reaction. The binding was also performed with GST-Sec3N, His6-Sec9C and His6-Sso2 under three different conditions: a: Sec3N, Sso2 and Sec9C were incubated with each other simultaneously; b: Sec3N and Sso2 were pre-incubated for 30 min at the room temperature before mixed with Sec9C; c: Sso2 and Sec9C were pre-incubated at room temperature for 2 h and then mixed with Sec3N. Proteins bound to GST-Sec3N were resolved by SDS–PAGE and detected by Coomassie blue staining.

Given that Sec3N directly interacts with Sso2, we tested whether Sec3 affects the formation of the binary Sso2-Sec9 t-SNARE complex. GST-tagged Sec9 C-terminal SNARE domain (a.a. 414–651) (‘Sec9C') was mixed with either His6-Sso2 alone or His6-Sso2 that was pre-incubated with Sec3N. The binding of Sso2 to Sec9C was analysed by SDS–polyacrylamide gel electrophoresis (SDS–PAGE). Sec9C and Sso2 alone were found to form a complex slowly (Fig. 2a, top panel). Addition of Sec3 significantly accelerated this interaction (Fig. 2a,b). The rate constant for the Sec9C–Sso2 interaction is 15.2±0.6 M^−1^s^−1^. The rate constant for the reaction with Sec3N pre-incubation is 200.3±90 M^−1^s^−1^. We noticed that Sec3N dissociated from the assembled t-SNARE complex (Fig. 2a, bottom panel). To further examine the relationship among these proteins, we immobilized GST-Sec3N on glutathione Sepharose, and tested its binding to Sso2 in the absence or presence of Sec9C. GST-Sec3N bound directly to Sso2, but not to Sec9C (Fig. 2c). Sec3N binding to Sso2 was lost when Sec9C was added in the binding reaction in different orders tested. Our results suggest that while Sec3N promotes Sso2–Sec9C interaction; Sec9C later displaces Sec3N from Sso2. The result also suggests that Sec3 does not directly affect later v-SNARE interaction as it is no longer in the Sso2–Sec9 binary complex.

### Sec3 stimulates SNARE-mediated liposome fusion

**Figure 3:**
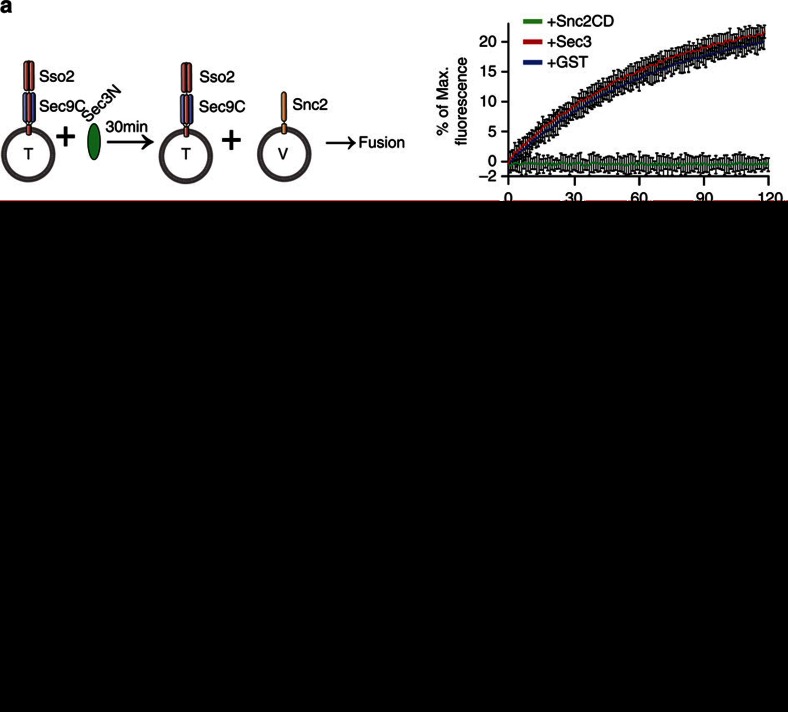
Sec3 stimulates SNARE-mediated liposome fusion. (**a**) The t-SNARE liposomes reconstituted with pre-assembled Sso2–Sec9C complex were incubated with Sec3N or GST (as a control) for 30 min. The v-SNARE liposomes incorporated with Snc2 were then added for the fusion reaction. Addition of Snc2 cytoplasmic domain (‘Snc2CD') inhibited fusion. The graphs represent the results of three experiments. Student's *t*-test was used for statistical analyses at 30, 60 and 90 min points. No statistical difference was found comparing pre-incubations with Sec3N and with GST. Error bars, s.d. (**b**) The t-SNARE liposomes reconstituted with Sso2 were pre-incubated with Sec3N or GST for 30 min. Sec9C and the v-SNARE liposomes incorporated with Snc2 were then added for the fusion reaction. Addition of Snc2CD inhibited the fusion reaction. The graphs represent the results of three experiments. Student's *t*-test was used for statistical analyses between pre-incubations with Sec3N and with GST at 30, 60 and 90 min points. *P*<0.01 for all these points. Error bars, s.d. (**c**) Liposomes containing Sso2 were incubated with Sec9C in the presence or absence of Sec3N at 25 °C for 30 min. The liposomes were collected by sedimentation. Proteins on the liposomes were separated by SDS–PAGE and detected by Coomassie blue staining. Addition of Sec3N led to increased association of Sec9C on Sso2 liposomes (comparing the second and third lanes). Also, less Sec3N remained on the Sso2 liposomes when Sec9 was added (comparing the third and fourth lanes). As a negative control, the association of Sec3N or Sec9C with Sso2-free liposomes was not detectable by Coomassie blue staining (the last two lanes). (**d**) Quantification of the amount of Sec9C bound to Sso2 liposomes in the presence or absence of Sec3N. The amount of Sec9 bound to Sso2 liposome in the absence of Sec3N was normalized to 1. Student's *t*-test was used for statistical analyses (*n*=3). Error bar, s.e.; ‘*'*P*<0.01).

As Sec3 directly interacts with Sso2 and promotes the Sso2–Sec9 binary complex formation, we speculate that Sec3 stimulates SNARE-mediated membrane fusion. We tested this hypothesis using *in vitro* fusion assays. The experiment was first carried out using liposomes, into which both Sso2 and Sec9C had been incorporated. Fusion was monitored as the de-quenching of NBD (N-7-nitro-2,1,3-benzoxadiazole) fluorescence from rhodamine-conjugated phospholipids as previously described^21^. Under this condition, Sec3N had no effect on the rate of fusion (Fig. 3a, compare red and blue lines). As a control, addition of the dominant-negative cytoplasmic domain of the Snc2 inhibited the fusion reaction (Fig. 3a, green line). Next, we performed the fusion assay using liposomes containing Sso2 alone. The liposomes were incubated with either Sec3N or GST before the addition of Sec9C and liposomes containing Snc2. The fusion reaction was slower than that with liposomes already incorporated with both Sso2 and Sec9C (Fig. 3b, blue line). Pre-incubation with Sec3 significantly accelerated membrane fusion (Fig. 3b, red line). This is consistent with the finding that Sec3 promotes Sec9–Sso2 interaction. The effect of Sec3 was not caused by liposome leakage as verified using the content-mixing assay with sulforhodamine B^22^ (Supplementary Fig. 2).

We have also performed fusion assays using an Sso2 fragment (‘Sso2CT', a.a.186–295), where the N-terminal inhibitory Habc domain is removed. The fusion rate was comparable to the full-length Sso2 pre-assembled with Sec9CT. Sec3N has no further stimulatory effect on Sso2CT-mediated fusion (Supplementary Fig. 3). These data is consistent with the model that Sec3 promotes fusion through relieving the auto-inhibition of Sso2.

We tested whether Sec3 promoted Sso2–Sec9C interaction on liposomes. Addition of Sec3N led to increased association between Sso2 and Sec9C (Fig. 3c, lane 2 and 3). On the other hand, we noted that less Sec3N remained on the liposomes when Sec9C associated with Sso2-liposomes (Fig. 3c, lane 3). This is consistent with the observation that Sec9C displaced Sec3 upon binding to Sso2 (Fig. 2). Altogether, the results are consistent with the hypothesis that Sec3 functions at the binary t-SNARE assembly stage.

### Crystal structure of the Sec3N–Sso2 complex

**Figure 4:**
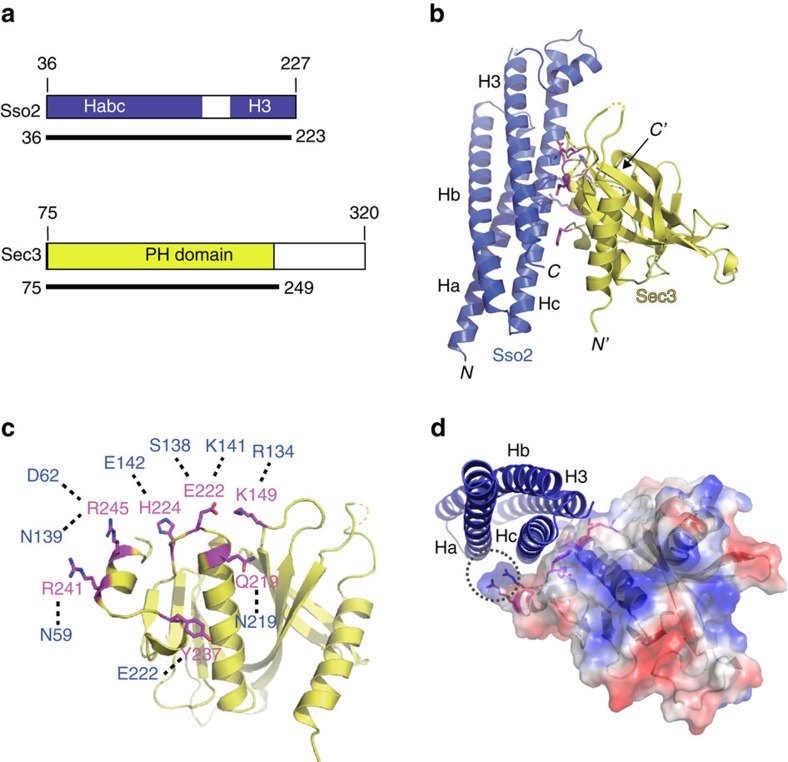
Crystal structure of the Sec3–Sso2 complex. (**a**) Schematics showing the constructs of Sso2 and Sec3 used in structural studies. Shown under the schematics are the boundaries of the visible parts of either protein in the final crystal structure. (**b**) Ribbon diagram of the crystal structure, with Sso2 and Sec3 shown in blue and yellow, respectively. Magenta sticks indicate the interface residues of Sec3 involved in the binary interaction. (**c**) Top view of the Sso2-binding sites on Sec3 with the seven major interface residues shown as magenta sticks. Corresponding binding residues on Sso2 are shown in blue. Dashed lines show hydrogen bonds or salt bridges. (**d**) End view of the Sso2 helical bundle showing that the binary interaction is mainly via the docking of Hc of Sso2 onto a cradle-like surface of Sec3, which consists of a relatively flat platform and a finger-like extension (dotted oval) to curve around the Hc motif.

To further understand the role of Sec3 in t-SNARE assembly, we crystallized Sec3N (a.a. 75–320) in complex with Sso2 (a.a. 36–227) (Fig. 4a,b). The structure was solved by molecular replacement using Sec3 (PDB code: 3A58) and Sso1 (PDB code: 1FIO) as searching models and refined to 2.20-Å resolution (Protein Data Bank code 5M4Y.pdb. Supplementary Table 1). Consistent with previously reports of Sec3 lipid-binding domain^23^,^24^, the core of Sec3N folds into a PH domain that contains a β-barrel formed by two sets of β-sheet with a helix capping one end (Fig. 4b,c). Sso2 is ordered as an anti-parallel four-helix bundle, with the region (a.a. 156–176) between its Habc domain (a.a. 36–155) and part of the SNARE motif (a.k.a. H3 domain) (a.a. 189–223) protruding aside (Fig. 4b). Part of the linker region (a.a. 177–186) and the C-terminus (a.a. 224–227) of Sso2 are not built due to poor electron density. The interface between Sec3N and Sso2 is ∼700 Å^2^, which is mediated primarily by 13 hydrogen bonds and 10 salt bridges (Fig. 4c and Supplementary Fig. 4). The seven main interface residues on Sec3 consist of K149, Q219, E222, H224, Y237, R241 and R245, which together describe a horseshoe-like shape on a relatively flat surface of the Sec3 PH domain (Fig. 4c). These residues provide a platform to make extensive contacts with Hc of Sso2, and some contacts with the SNARE motif of Sso2 (Supplementary Fig. 4). Additionally, side chains of residues R241 and R245 of Sec3 form a finger-like structure that cradles around Hc and reach to the Ha motif of Sso2 (Fig. 4d).

**Figure 5:**
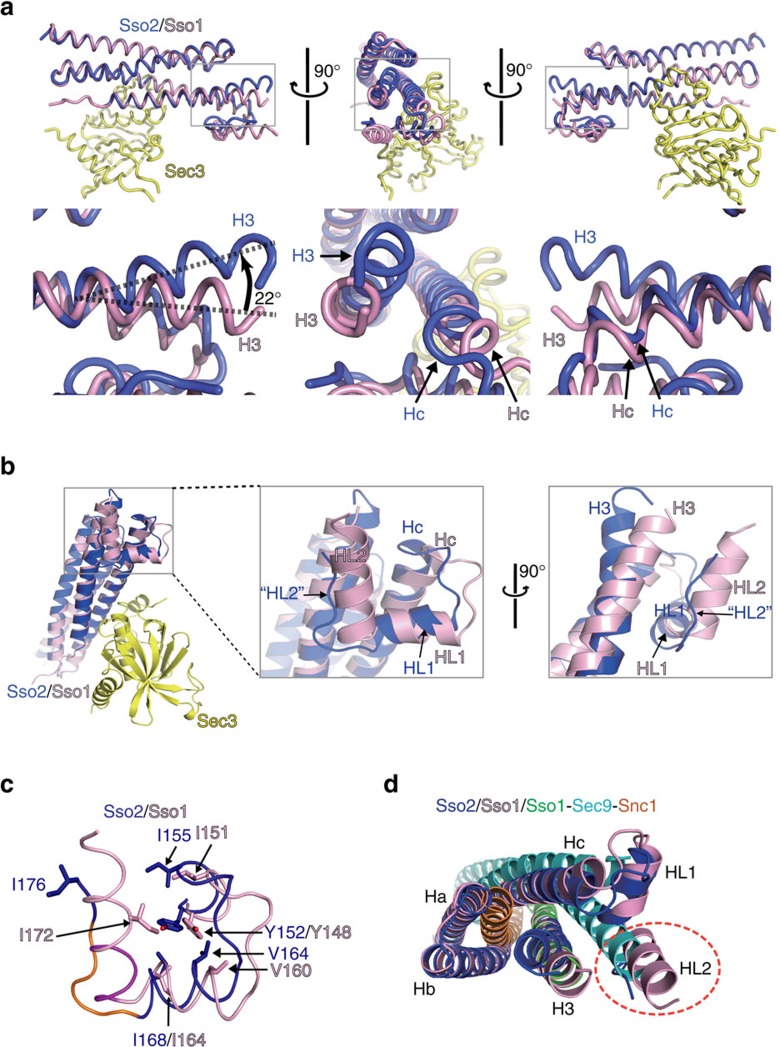
Structural changes of Sso2 induced by Sec3N. (**a**) Structural comparison of free Sso1 and Sso2 in complex with Sec3. Three orthogonal views are displayed to show the structural changes at the SNARE motif and Hc region. Boxed parts are enlarged under the images for a clear view of the structural changes. In comparison to Sso1, the N terminus of SNARE motif (‘H3') in Sso2 bends towards Ha by 22°. (**b**) Conformational transition of Helix 2 (‘HL2') in the linker region. ‘HL2' of Sso2 is mostly disordered when bound to Sec3N. (**c**) A close-up view of the conformational changes in the linker region. Residues forming the hydrophobic core are shown as sticks. (**d**) Superposition of Sec3-bound Sso2 (blue), free Sso1 (pink) and the Sso1-Sec9–Snc1 complex (green, cyan and orange; PDB code: 3B5N). The alignment was centered on the SNARE motif of Sso. Note that HL2 (dotted oval in orange) appears in a position that hinders Sec9 binding to Sso.

Previous studies have shown that Sso1 adopts a ‘closed' conformation when in isolation, with its SNARE motif packed against the N-terminal inhibitory Habc domain^6^,^8^. Sso2 and Sso1 are close paralogs in yeast that share a very high-degree of sequence identity (Supplementary Fig. 5), with many features shared structurally and conserved in mammalian syntaxins (see below)^2^,^3^,^25^. Significant structural differences between Sec3-bound Sso2 and isolated Sso1 are observed. In the Sso2-Sec3 crystal structure, the N terminus of the H3 domain bends approximately 22° away from its original position, and is tilted towards the Ha domain (Fig. 5a). There is a linker region between the N terminus of the SNARE motif and Hc (Fig. 5b). This region in isolated Sso1 consists of two helices, named HL1 (a.a. 156–164) and HL2 (a.a. 165–178), and is stabilized by a hydrophobic core formed by residues from HL1 and HL2 and the C terminus of the Hc^8^. In the Sso2–Sec3N complex, however, HL1(a.a. 160–168) is dislocated and HL2 turns into a mostly disordered loop (Fig. 5b,c). The crystal structure suggests that Sec3 interaction causes the bending of the SNARE motif, which then led to the disruption of the hydrophobic interactions in the core of Sso2 involving HL2. Such changes could facilitate the release of the SNARE motif of Sso2 from its Habc domain when Sec9 is available. Superimposition of the SNARE motifs from free Sso1 and SNARE complex shows that HL2 occludes a portion of the Sec9-binding interface^8^,^26^ (Fig. 5d).

Besides the C222_1_ crystal form described above, we have also obtained another crystal form that belongs to space group P4_3_2_1_2 and contains one Sec3/Sso2 dimer per asymmetric unit under a very different crystallization condition (Protein Data Bank code 5LG4.pdb. Supplementary Table 1, and see Methods). The four Sec3/Sso2 dimers in the two crystal forms are essentially identical (Supplementary Fig. 6a). Both the bending of the SNARE motif N-terminus and the relaxation of HL2 of Sso2 described above are found in all four copies of the Sec3/Sso2 complex present in the two different crystal forms (Supplementary Fig. 6b). These data strongly suggest that the structural changes around the linker region of Sso2 are induced upon Sec3 binding rather than from crystallization artifacts.

### Sso2 HL2 mutation promotes liposome fusion without Sec3

**Figure 6:**
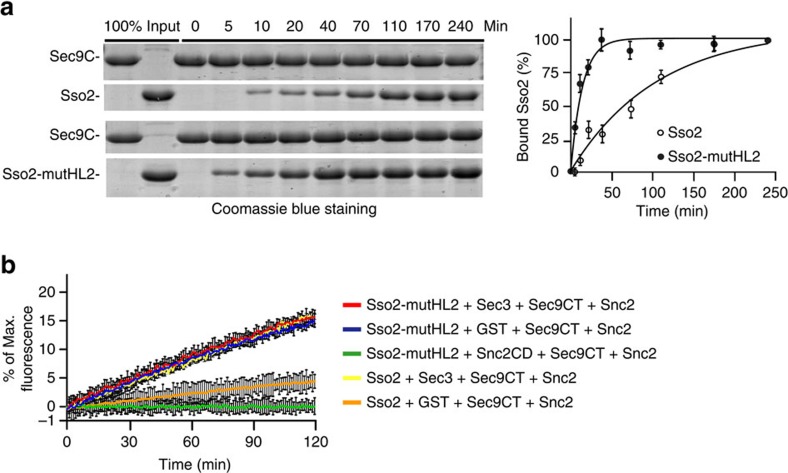
Mutation in Sso2 HL2 leads to faster t-SNARE assembly and liposome fusion. (**a**) His6-tagged Sso2 or *Sso2-mutHL2* was incubated with GST-Sec9C conjugated to glutathione Sepharose 4B for the binding assay. Samples were taken at indicated time points and analysed by SDS–PAGE and Coomassie blue staining. The bound Sso2 at different time points was quantified, with bound Sso2 at 240 min without Sec3N normalized as 100%. The percentage of binding was plotted. The points in the graph represent the average of three experiments. Student's *t*-test was used for statistical analyses. Error bars: s.e. ‘*'*P*<0.05. (**b**) The t-SNARE liposomes reconstituted with the wild-type Sso2 or Sso2-mutHL2 were pre-incubated with Sec3N or GST for 30 min. Sec9CT and the v-SNARE liposomes incorporated with Snc2 were then added for the fusion reaction. Addition of Snc2CD inhibited the fusion reaction. The graph represents the results of three experiments. Student's *t*-test was used for statistical analyses. No statistical significance was found comparing the fusion rates of Sso2-mutHL2 liposomes (no Snc2CD) and that of the wild-type Sso2 liposomes pre-incubated with Sec3N. Error bars, s.d.

To test whether disrupting the folding of HL2 facilitates Sso2–Sec9 interaction, we generated an Sso2 mutant (‘*sso2-mutHL2*': ‘^170^DVNGQ^174^' to ‘^170^GSSGG^174^') so that the HL2 region cannot form a stable α-helix structure. Our *in vitro* binding assay shows that Sso2-mutHL2 assemblies with Sec9 much faster than the wild-type Sso2 (Fig. 6a). We next performed fusion assays using liposomes incorporated with Sso2-mutHL2. The fusion rate of Sso2-mutHL2 liposomes (Fig. 6b, blue line) was comparable to that with the wild type Sso2 pre-incubated with Sec3 (Fig. 6b, yellow line). Pre-incubation of Sso2-mutHL2 with Sec3 did not further accelerate liposome fusion (Fig. 6b, red line). Taken together, the data suggest that the dissolving of HL2 facilitates t-SNARE assembly and membrane fusion.

### Sec3–Sso2 complex disruption leads to defective exocytosis

**Figure 7:**
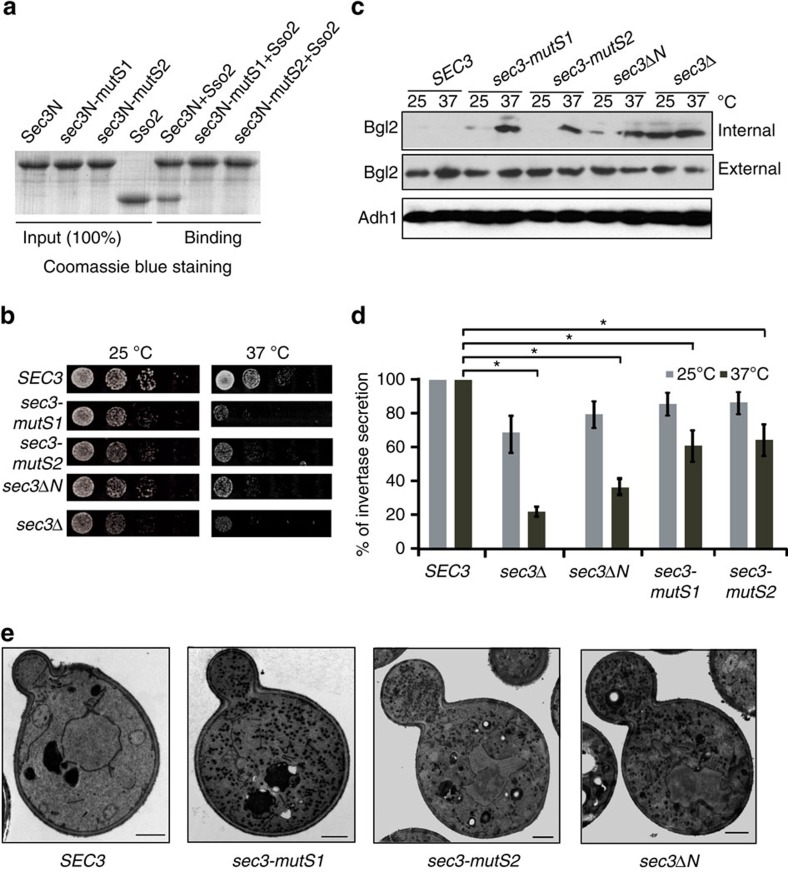
Disruption of the Sec3–Sso interaction in yeast leads to exocytosis defects. (**a**) His6-Sso2 was incubated with GST-tagged wild-type or mutant Sec3N for binding assay. The *sec3N-mutS1* and *sec3N-mutS2* mutants showed much reduced binding to Sso2. (**b**) *SEC3*, sec3Δ*N, sec3-mutS1* and *sec3-mutS2* were expressed under the endogenous *SEC3* promoter in the *sec3*Δ background. The yeast cells were grown on synthetic complete plates at 25 and 37 °C. The *sec3* mutants show growth defects at 37 °C. (**c**) Analysis of Bgl2 secretion for the above yeast strains. Yeast cells were grown at 25 °C before being transferred to 37 °C for 2 h. Internal and external Bgl2 was detected by western blotting. Alcohol dehydrogenase-1 (Adh1) was used as a loading control. (**d**) Invertase secretion from yeast cells of the above strains at 25 and 37 °C. The percentage of invertase secretion was plotted. The values in the graph represent the average of three experiments. Student's *t*-test was used for statistical analyses. Error bars: s.e. ‘*'*P*<0.05. (**e**) Thin-section electron microscopy analysis of cells expressing *SEC3, sec3*Δ*N*, *sec3-mutS1* and *sec3-mutS2*. Cells were fixed with permanganate, and the vesicles were shown in dark staining. An accumulation of secretory vesicles was detected in cells expressing Sec3 mutants. Scale bar, 0.5 μm.

To examine the functional significance of the Sec3–Sso2 interaction, we mutated seven (‘*sec3-mutS1*': K149E, Q219K, E222K, H224D, Y237D, R241E, R245E) or four (‘*sec3-mutS2*': K149E, E222K, H224D, Y237D) residues in the Sso2 contact region of Sec3. The mutagenesis did not cause overall structure changes of Sec3N as confirmed by our biophysical experiments including analytical SEC, thermofluor assay and circular dichroism (Supplementary Fig. 7). Both mutants have much reduced interaction with Sso2 compared with the wild-type Sec3 (Fig. 7a). Sec3 is involved in targeting vesicles to the plasma membrane through its interaction with PI(4,5)P_2_ and GTP-Cdc42 (refs 23, 27, 28, 29). Our binding assay shows that mutations in *sec3-mutS1* do not affect the interactions of Sec3 with either PI(4,5)P_2_ or Cdc42 (Supplementary Fig. 8). Also, the mutant Sec3 protein remains polarized to the daughter cells as determined by fluorescence microscopy (Supplementary Fig. 9). These results are consistent with our combinatorial structure analysis, which shows that Sec3N binding to Sso2 is separate from its interactions with PI(4,5)P_2_ and Rho1 (another member of the Rho family of small GTPases that share almost identical Sec3-binding motif as Cdc42) (Supplementary Fig. 10). All of these data suggest that the Sec3-Sso binding interface is separated from the Sec3 membrane-targeting domain.

Next, we examine the effect of Sec3 mutations on exocytosis. *sec3-mutS1, sec3-mutS2* and Sec3 with its N-terminal 320 amino acids deleted (‘*sec3*Δ*N*') were expressed under the control of the *SEC3* promoter to replace the endogenous *SEC3* in yeast. The growth of these mutants was examined on synthetic complete plates. *sec3*Δ and *sec3*Δ*N* strains were viable at 25 °C, but barely survived at 37 °C (Fig. 7b), consistent with the previous observations^29^,^30^. Similarly, *sec3-mutS1* and *sec3-mutS2* showed poor growth at 37 °C (Fig. 7b). To examine exocytosis, *sec3* mutant cells were grown at 25 °C, and then shifted to 37 °C for 2 h. The *sec3-mutS1, sec3-mutS2* and *sec3*Δ*N* cells accumulated Bgl2, a cell wall modification enzyme, inside the cells (Fig. 7c and Supplementary Fig. 11a). In addition, the secretion of the periplasmic enzyme invertase was also affected (Fig. 7d). Thin-section electron microscopy shows that the *sec3-mutS1*, *sec3-mutS2* cells accumulate electron-dense vesicles of 80-100 nm in diameter, the characteristic size of post-Golgi secretory vesicles (Fig. 7e). Finally, Sec3N-mutS1 failed to stimulate liposome fusion (Supplementary Fig. 11b). Taken together, the data suggest that disruption of the Sec3–Sso2 interaction inhibits exocytosis.

### Separate Sec3 functions in fusion and vesicle tethering

**Figure 8:**
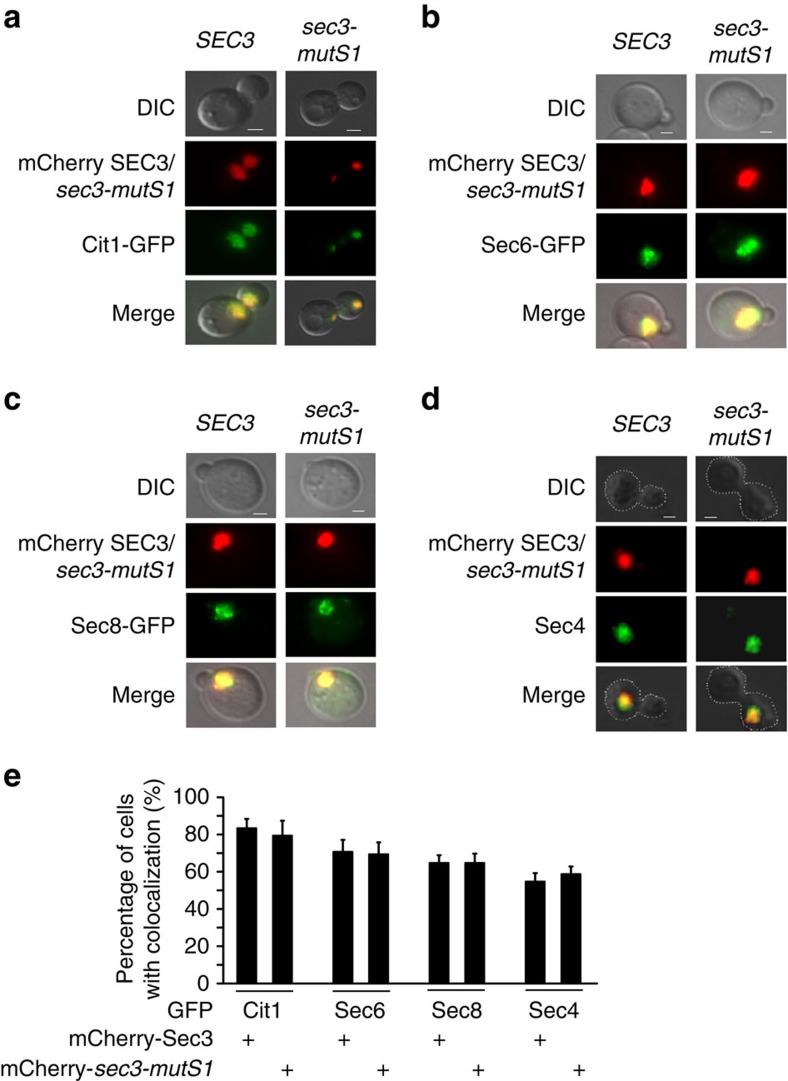
The Sec3 Sso2-binding mutant is capable of tethering vesicles. (**a**) Co-localization of Tom20-mCherry-*SEC3* or Tom20-mCherry-*sec3-mutS1* with Cit1-GFP, a marker for yeast mitochondria. Scale bar, 1 μm. (**b**) Ectopically targeted Tom20-mCherry-*SEC3* and Tom20-mCherry-*sec3-mutS1* were both able to recruit Sec6-GFP to mitochondria clusters. (**c**) Ectopically targeted Tom20-mCherry-*SEC3* and Tom20-mCherry-*sec3-mutS1* were both able to recruit Sec8-GFP to mitochondria clusters. (**d**) Yeast cells were fixed and spheroplasted for immunofluorescence staining of the Rab protein Sec4, a marker for post-Golgi secretory vesicles. Sec4 vesicles were recruited to mitochondria. (**e**) Quantification of yeast cells expressing Sec3 or *sec3-mutS1 with* mitochondria-exocyst co-localization and mitochondria-Sec4 co-localization. Three independent experiments were performed. In each experiment, 50 cells were counted. The percentage of cells with co-localization was plotted. Student's *t*-test was used for statistical analyses. Error bars: s.e. No statistical significance was found comparing Sec3 and *sec3-mutS1.*

The exocytosis block in Sec3 mutants could also result from a defect in vesicle tethering. To test this possibility, we adopted an ectopic targeting strategy recently developed in the field^31^,^32^,^33^. It was shown previously that Sec3, when tagged with Tom20, could be targeted to mitochondria clusters in yeast cells; the ectopically localized Sec3 then recruited the other exocyst subunits and tethered secretory vesicles to these mitochondria clusters^32^. Here we targeted *sec3-mutS1* in yeast cells using the same strategy. Similar to the wild-type Sec3, *sec3-mutS1* was targeted to mitochondria clusters (Fig. 8a). Following Sec3 or *sec3-mutS1*, Sec6 and Sec8, two components of the exocyst, and Sec4, the exocytic Rab protein and a marker for post-Golgi secretory vesicles, were ectopically recruited to mitochondria (Fig. 8b–d). Cells expressing Sec3 and *sec3-mutS1* show similar levels of mitochondria-exocyst colocalization or mitochondria-Sec4 co-localization (Fig. 8e). This result suggests that *sec3-mutS1* retains its ability to tether secretory vesicles, and is in agreement with our previous study that Sec3 interacts with and recruits other exocyst subunits through its C terminus^29^,^32^. Our result also suggests that the role of Sec3 in promoting SNARE-mediated fusion is separable from its previously demonstrated role in vesicle tethering.

## Discussion

To date, a number of studies, especially those from the field of neurotransmission, have contributed tremendously to the understanding of SNARE assembly^34^,^35^. However, mechanisms that control the initial binary t-SNARE formation remain elusive. In many cell types the t-SNAREs are not pre-assembled. For example, in yeast, the majority of the t-SNARE proteins Sso and Sec9 are not in a complex with each other^20^. In mammals, syntaxins and SNAP-25 only partially co-localize at the plasma membrane^36^,^37^. Factors promoting the initial t-SNARE interaction are clearly needed in cells.

Here we find that Sec3 promotes the assembly of the Sso2 and Sec9 binary t-SNARE complex, the first rate-limiting step in SNARE assembly during exocytosis^4^,^8^. Our liposome reconstitution assay demonstrates that Sec3 promotes fusion at a step before the Sec9–Sso2 interaction. Crystal structure shows that Sec3 binds to the Hc and SNARE motif of Sso2, and causes 22° bending of the N terminus of the SNARE motif. The bending renders significant conformational changes at a region that links these two domains. This region, consisting of HL1 and HL2, form a hydrophobic core that stabilizes the closed conformation of isolated Sso2. In fact, HL2 occludes a significant portion of Sec9/SNAP-23-binding site on H3 for both yeast and mammalian SNAREs^8^. The relaxation of the HL2 helical structure upon Sec3 binding disrupts the stability of the closed conformation of Sso2, and poises Sso2 for subsequent Sec9 binding. Supporting this model, our *in vitro* binding experiment shows that Sso2 with mutations that disrupt helix formation in HL2 has faster rate of assembly with Sec9 than the wild-type Sso2. Liposomes incorporated with the Sso2 HL2 mutant are faster than those with wild-type Sso2 in fusion. Interestingly, it was previously shown that mutations in Sso1 in the hydrophobic region, including I172A located in the HL2 of Sso1, destabilized the hydrophobic core and markedly accelerated the interaction between Sso1 and Sec9 (ref. 38). In mammals, the L165A/E166A mutations in the linker region of syntaxin-1a promoted its transition from the ‘closed' to ‘open' state^5^. It is noteworthy that the bending of the SNARE motif was not observed in either free Sso1 or the assembled SNARE complex^2^,^8^,^26^. It is possible that Sec3 binding renders Sso2 to an intermediate state between the free and assembled SNARE, in which the SNARE motif is poised to dissociate from the inhibitory Habc domain when Sec9 is available. After Sec9 binding, Sec3 is displaced from the t-SNARE complex so that the final v- and t-SNARE complex can assemble. Based on the crystal structure, Sec3N has a PH-domain like structure that is well conserved from yeast to plants and human^23^,^24^. It will be interesting to test whether the Sec3–SNARE interaction is conserved in other eukaryotic cells.

The exocyst belongs to the CATCHR family of multi-subunit tethering proteins^14^. These proteins may promote fusion by physically bringing the vesicles and the target membrane together before the action of the SNAREs, a function consistent with the concept of ‘tethering'. For example, the Dsl1 complex forms a long ‘tower'-like structure that interacts with the t-SNAREs at the ER and the COPI vesicles^39^,^40^; Dsl1 may promote v- and t-SNARE assembly by capturing the vesicles. During homotypic vacuolar fusion in yeast, the HOPS complex may promote fusion by tethering vacuoles and by the recruitment of the soluble SNARE Vam7 to the vacuolar membrane^41^,^42^. Similarly, the exocyst may also contribute to fusion by ‘tethering'. Sec6 has been found to interact with both Sec9 and the v-SNARE protein Snc1/2 (refs 43, 44). However, Sec6 does not have any stimulatory effect on SNARE assembly or membrane fusion^43^,^44^. A recent study showed that Sec6 binds to fully assembled SNAREs^45^. Here we propose a different model, in which Sec3 affects fusion by catalysing the binary t-SNARE interaction. This function is mediated by the N terminus of Sec3, which is separate from the C-terminal domain that mediates exocyst complex assembly and vesicle tethering^29^,^32^. Furthermore, disruption of the Sec3–Sso2 interaction does not affect its ability to interact with Cdc42 and PI(4,5)P_2_, and its polarized localization to the bud tip. Ectopic tethering assay using mitochondria as a surrogate target organelle demonstrates that the Sso-binding-deficient *sec3* mutant retains its ability to recruit other exocyst subunits as well as the secretory vesicles. Our data suggest that the function of Sec3 in promoting fusion is distinct from its role in vesicle tethering. In Golgi, it was previously reported the tethering protein p115 promotes SNARE assembly^46^,^47^,^48^. It would be interesting to examine whether such a ‘catalytic' role in membrane fusion exist for tethers in other stages of vesicular trafficking.

Besides the exocyst, the Sec1/Munc18 (SM) family of proteins also has important roles in SNARE-mediated fusion, although different models have been proposed, depending on the cell type and stage of membrane trafficking^49^,^50^. A recent study showed that SM protein Sly1 accelerates the ER-Golgi SNARE assembly^10^. During homotypic fusion of yeast vacuoles, Vps33, the SM protein and a member of the HOPS tethering complex, binds to SNARE proteins in each vacuole, and registers them in the correct orientation for subsequent SNARE complex assembly^51^. Sec1 in yeast binds to the fully assembled SNARE complex^52^; genetics analyses suggest that Sec1 functions both before and after SNARE assembly^53^. Genetic analyses also place Sec1 at a step after the exocyst^54^,^55^,^56^, and it was shown that Sec1 binds to the exocyst^57^. It is, therefore, possible that Sec3 first acts on Sso2 on its auto-inhibitory state and promotes the SNARE assembly before the action of Sec1.

Beside the SM family, proteins with the MUN domain were also shown to regulate SNARE assembly during synaptic transmission^58^,^59^. It was proposed that, during neuronal exocytosis, Munc13 relieves the inhibition of syntaxin-1 imposed by the SM protein Munc18 (ref. 60). In yeast, Sec3 does not work through a similar mechanism as observed in neurotransmission because Sec1 binds to Sso2 only when it is in the later fully assembled ternary SNARE complex^49^. However, some members of the exocyst contain regions that are structurally similar to the MUN domain^61^,^62^. It is, therefore, possible that, in addition to Sec3, other members of the exocyst also contribute to SNARE assembly during exocytosis. In support of this possibility, *sec3ΔN* displays defects in exocytosis at 37 °C, but the defects is not clear at 25 °C (ref. 29 and this study), and *sec3ΔN* is synthetic lethal with a number of mutants including *exo70* (refs 28, 63, 64). It will be interesting to examine the role of other exocyst subunits in SNARE assembly and membrane fusion.

## Methods

### Plasmids and yeast strains

All mutagenesis experiments were carried out using a multi-mutagenesis kit (Agilent Technologies). All plasmids used in this paper are listed in Supplementary Table 2. Sec3 mutants were made from *CEN-LUE2* plasmid (pG1273), which contains full-length Sec3 and its promoter and terminator sequences. The Sec3 N-terminal (a.a. 1–320) region was sub-cloned into a GST fusion protein vector (pGET-4T-1). His6-Sso2 (aa.1–270) (pG707) and His6-Sec9C (a.a. 414–651) (pG1509) were subcloned from GST-Sso2 (a.a. 1–270) (pG638) and GST-Sec9C (a.a. 414–651) (pG1067). Mutant Sec3-GFP plasmid (pG1926) was derived from a Sec3-GFP plasmid (pNB810) (ref. 65). His6-Sso2-mutHL2 (pG1971) was directly mutated from pG707. pG1973 and pG1974 were derived from pG707 and pG1971. SNAREs used in liposome mixing assay were kindly provided by Dr James McNew (Rice University). To generate expression constructs for structural studies, sequences encoding the Habc and the SNARE motif of Sso2 (a.a. 36–227) and the Sec3 N-terminal domain (a.a 75–320) were cloned into pET15b (Novagen) and the custom vector MalpET, respectively. Vector pET15b provides a thrombin-cleavable N-terminal His6 tag, whereas MalpET supplies an N-terminal tobacco etch virus protease-cleavable MBP-His10 tag. All yeast strains used in this project are listed in Supplementary Table 3. Yeast growth and genetic manipulations were carried out using standard procedures.

### Yeast cell extract preparation

Yeast cells were grown to an OD_600_ of 1.0 and collected by centrifugation. The pellet was washed and then resuspended in ice-cold lysis buffer (50 mM Tris-HCl, pH 7.5, 100 mM NaCl, 1 mM EDTA, 1% Triton X-100, 5 mM NaF, 1 mM sodium pyrophosphate, 1 mM dithiothreitol, and protease inhibitor cocktail; Roche). Glass beads (0.4–0.6 mm in diameter) were added to the resuspended cells. Cells were broken by vigorous vortexing. The beads and cell debris were removed by centrifugation at 13,000*g* for 30 min at 4 °C.

### Protein expression and purification

The *E. coli* BL21(DE3) cells harbouring the expressing plasmids were cultured in Luria broth medium containing ampicillin or kanamycin at 37 °C to an OD_600_ of 0.6–1.0. Protein expression was induced with 0.5 mM isopropylthio-β-d-galactoside at 18 °C overnight. The cells were then harvested by centrifugation and lysed by sonication. After removal of cellular debris by centrifugation at 13,000 r.p.m. at 4 °C for 30 min, the supernatant fraction was extracted with 2% (for proteins with trans-membrane domains) or 0.5% (all other proteins) Triton X-100 for 1 h at 4 °C. Glutathione Sepharose 4B or Metal Affinity Resin (Clonetech) were added to bind GST-tagged or His6-tagged proteins for 2 h at 4 °C. After washing three times, bound proteins were eluted by 10 mM glutathione or gradient imidazole (25–200 mM) and dialysed in PBS buffer.

To carry out protein expression for structure studies, *E. coli* BL21(DE3) cells were transformed with plasmid DNA and grown in Luria broth medium at 37 °C to an OD_600_ of 0.6–0.8. Recombinant protein expression was induced by addition of 0.5 mM isopropylthio-β-d-galactoside followed by an incubation for 14 h at 18 °C. Cells were harvested by centrifugation (6,000 *g*, 15 min) and pellets were resuspended in cold lysis buffer (20 mM Tris-HCl pH 8.0, 250 mM NaCl, 20 mM imidazole, 5% (v/v) glycerol, 5 mM β-mercaptoethanol) supplemented with 1 mM PMSF and 20 μg ml^−1^ DNase I. Resuspended cells were lysed using an EmulsiFlex-C3 homogenizer (Avestin) and cell debris were removed by centrifugation (16,000*g*, 40 min). Supernatants were filtered through a 0.45-μm filter and applied onto a 5-ml Ni-HiTrap column (GE Healthcare) pre-equilibrated in the same lysis buffer. Bound proteins were eluted with a linear concentration gradient of 20–600 mM imidazole in the lysis buffer.

The MBP-10 × His tag of Sec3 was cleaved off by tobacco etch virus protease and then removed using an anion exchange column (HiTrap-Q HP, GE Healthcare). The Q column was pre-equilibrated with a buffer containing 20 mM Tris-HCl pH 8.0, 50 mM NaCl, 5% (v/v) glycerol, and 2 mM dithiothreitol. The His6 tag of Sso2 was removed by thrombin and then purified on the HiTrap-Q HP column. Both proteins were purified to homogeneity using a Superdex-200 16/60 column (GE Healthcare) pre-equilibrated with a buffer containing 20 mM Tris-HCl pH 8.0, 100 mM NaCl, 5% (v/v) glycerol, and 2 mM dithiothreitol.

To prepare the Sec3/Sso2 complex, purified Sso2 were mixed with Sec3N in excess and then left at 4 °C overnight. The reconstituted complex was purified using a Superdex-200 16/60 column (GE Healthcare). Elution fractions were examined by SDS–PAGE and those containing the protein complex were pooled and concentrated to 15 mg ml^−1^. Portions of the protein sample was used directly for crystallization.

### Binding assays

Sec3 N-terminus (pG1860, a.a. 1–320, Sec3N) and Exo70 C-terminus (pG276, a.a. 358–623, Exo70C) were expressed as GST fusion proteins. The cytosolic domains of Sso2 (a.a. 1–270, His6-Sso2) and Pep12 (a.a. 1–263, His6-Pep12) were expressed as His6-tagged fusion proteins. Purified Sec3NT and Exo70CT (12 μg) were coupled to glutathione Sepharose 4B. His6-Sso2 (2 μM final concentration) and His6-Pep12 (4 μM final concentration) were then added for incubation at 25 °C for 2 h. After three washes in PBS buffer, fractions of the protein samples bound to the Sepharose were analysed by SDS–PAGE and detected by Coomassie blue staining. The binding of sec3-mutS1 and Sso2 was performed the same as described above. To test full-length Sec3 interaction, Sec3 was inserted into the pcDNA3 expression vector (Invitrogen) for *in vitro* transcription/translation in the presence of [^35^S] methionine in rabbit reticulate lysates (Promega). Twenty microlitres of lysates was used in a binding reaction with final concentration 2 μM GST-tagged cytosolic domains of Sso2 (a.a. 1–270) or Pep12 (a.a. 1–265). In all, 2.5% of the total input and the bound Sec3 were analysed by SDS–PAGE and autoradiography.

To test the effect of Sec3 on the Sso2–Sec9 interaction, Sec9 C terminus (pG1067, a.a. 414–651, ‘Sec9CT') was expressed as a GST fusion protein. Cytosolic domains of Sso2 (a.a. 1–270) and Sec3N were expressed as His6-tagged fusion proteins. His6-Sso2 (2 μM final concentration) was incubated with His6-Sec3N (2 μM final concentration) or an equal amount of buffer for 30 min at 25 °C to allow the formation of the Sso2–Sec3 complex, and then added to GST-Sec9C fusion protein (20 μg) conjugated to glutathione Sepharose 4B at a final volume of 100 μl. Fractions of the reaction mixture were taken at indicated time points to analyse the interactions. To test the displacement of Sec3 from Sso2, 5 μg of His6-tagged Sec9C was used.

### Quantification of Coomassie blue-stained gel

Quantification of Coomassie blue-stained gels was performed with the ImageJ software. The bands of interest were manually circled. The area and average intensity of the circled bands were calculated by the software. Similar circles in the adjacent area without bands were selected as control. Its average intensity was also measured, and later subtracted. Each binding point in the graph represents the average of three experiments. Student's *t*-test was used for statistical analyses.

### Kinetics analysis

The method to calculate rate constant, *k*, for the formation of t-SNARE complex was previously described^4^. Briefly, the equation θ(*t*) =θ_0_+(θ_∞_−θ_0_)(*A*_0_*kt*)/(*A*_0_*kt*+1) is used to fit the curve. θ(*t*) is normalized value of bound Sso2 band intensity at time *t*, θ_0_ is zero here as no Sso2 was bound at *t*=0, θ_∞_ is the band intensity at *t*=∞ and is subjected to fitting together with apparent rate constant *k*, *A*_0_ is the initial concentration of Sso2 in molar units (M), *k* is the rate constant in M^−1^s^−1^, and *t* is the time in seconds since the start of mixing.

### Crystallography and structure determination

Initial crystallization screenings were carried out at 22 °C by the sitting drop vapor diffusion method using the Phoenix HT liquid handling robot (Rigaku) to set-up dual droplets for each condition with drop volume of 0.2 and 0.3 μl (1:1 and 2:1, protein vs reservoir solution, respectively) on the 96-well sitting drop crystallization plate (Molecular Dimensions). Final crystals used for data collections were grown from manually set up drops containing l μl of protein and l μl of reservoir solution. Plate-like crystals of ∼10 × 100 × 100 μm (crystal form 1, space group C222_1_) were obtained in a condition containing 0.1 M HEPES (pH 7.0), 0.2 M NaCl, and 25% (w/v) PEG 3350, whereas rod-like crystals of ∼20 × 20 × 100 μm (crystal form 2, space group P4_3_2_1_2) were obtained in a condition containing 0.1 M Tris-HCl (pH 8.5) and 2 M (NH4)_2_SO_4_ (all chemicals were from Sigma Aldrich). Both crystals appeared after 2–3 days and reached maximal size after approximately 2 weeks. The crystals were harvested with 20 micron diameter nylon loops (Hampton Research) by flash-cooling in liquid nitrogen using the same reservoir solutions containing 20% (v/v) glycerol as the cryo-protectant. Shutterless data collections for both crystal forms were carried out by a continuous rotation around the Phi axis with a fast readout Pilatus 6M pixel detector (Dectris LTD), following the strategies suggested by the EDNA program implemented in mxCuBE at the beamline ID29 of the European Synchrotron Radiation Facility in Grenoble, France, with a selected beam size of 30 μm in diameter.

Data reduction was carried out using the XDS program^66^. For structure determination, the maximum-likelihood molecular replacement with PHASER^67^ was carried out using the previously determined structures of Sec3 (PDB code: 3A58) and Sso1 (PDB code: 1IFO) as the searching models^8^,^24^. The resulting models were checked and manually rebuilt using the program COOT^68^. Refinement was carried out by Phenix.refine^69^ using data of 20-2.20 Å (crystal form 1) and 20-2.90 Å (crystal form 2), but using NCS restraints in the refinement only for the former. All subsequent structure analyses and figure generations were carried out by Pymol ( http://www.pymol.org). The details of data collection and refinement statistics are summarized in Supplementary Table 1.

### Thermofluor assays

The assay was carried out based on a previously published protocol^70^. 5,000 × stock solution of Sypro Orange dye (Molecular Probes) was diluted by 300-fold in water. Solutions of 12 μl of protein buffer (20 mM Tris-HCl, pH 8.0, 100 mM NaCl), 8 μl of Sec3 or *Sec3-mutS1* (2.5 mg ml^−1^) and different amounts of Sypro Orange dye (1, 2, 3, 4 or 5 μl adjusted with water to 5 μl in total) were added onto a 96-well thin-wall PCR plate (Bio-Rad). The plate was then sealed with optical-quality sealing tape (Bio-Rad) and heated in an iCycler iQ real-time PCR detection system (Bio-Rad) from 15 to 95 °C with increments of 0.5 °C per 10 s.

### Circular dichroism

Circular dichroism measurements were carried out on a Chirascan-plus unit (Applied Photophysics) with a 0.5-mm path-length cuvette at 25 °C. Protein samples of 0.2 mg ml^-1^ were dissolved in 20 mM Na_2_HPO_4_/NH_2_PO_4_ pH 8.0. Spectra from three consecutive scans (260–180 nm, 0.5 s averaging time, 0.5 nm steps) were averaged. Data points were exported and curves were generated using Windows Excel-2013 (Microsoft).

### Proteoliposome fusion assays

The SNARE proteins (Sso2, Sec9, Snc2) used in this study were described previously^21^. For t-SNARE proteoliposomes 100 μl of Sso2 (10 mg ml^−1^) with Sec9CT (10 mg ml^−1^) or an equal amount of buffer were mixed at 4 °C overnight in 1% n-octyl-β-d-glucopyranoside. Lipids of POPC/DOPS (85%:15%) were then mixed with Sso2 or Sso2/Sec9CT. For v-SNARE proteoliposomes, 50 μl of Snc2 (1 mg ml^−1^, cleaved from GST-Snc2) with 1% octyl-β-d-glucopyranoside was used to prepare a donor lipid mix with 82% POPC, 15% DOPS, 1.5% NBD-DPPE and 1.5% Rohdamine-DPPE. The octyl-β-d-glucopyranoside in lipid-protein mixture was adjusted to 1%. After vigorous vortexing, proteoliposomes were formed by diluting the lipids–protein mixture to final octyl-β-d-glucopyranoside concentration 0.33%. Then the detergent was removed by dialysis overnight. Proteoliposomes were isolated by flotation in a discontinuous Nycodenz gradient. To prepare sulforhodamine-loaded liposomes, SNARE liposomes were reconstituted by the same procedure above. In addition, buffer with 75 mM sulforhodamine B (Sigma, Inc.) was used to dilute SNARE-lipids mix to form liposomes. Free sulforhodamine B was removed by overnight dialysis. To perform the fusion assay, GST-Sec3N (5 μM) or GST (5 μM) was incubated with Sso2 incorporated or Sso2/Sec9CT incorporated t-SNARE at 25 °C, 30 min. Forty-five microlitre protein treated t-SNARE, Snc2 (5 μM) and Sec9CT (5 μM) were incubated at 37 °C for 2 min. The fluorescence signals were monitored by a Gemini EM Fluorescence Microplate Reader (Molecular Devices). Each point in the graph represents the average of three experiments.

### LUV sedimentation assay

The LUV sedimentation assay was performed as previously described^63^. The binding of Sec3N and Sec3N mutants to LUVs was determined by sedimentation assays conducted in 200 μl of total volume in an ultracentrifuge rotor (TLA-100; Beckman Coulter). The tubes were pre-incubated for 1 h in a 50- μM solution of PC in the HNa100 buffer to prevent nonspecific binding of Sec3 to polycarbonate centrifuge tubes. Sucrose-loaded LUVs were precipitated at 150,000*g* at 25 °C for 30 min. The pellets were subjected to 12% SDS–PAGE and stained with Coomassie blue. ImageJ software was used for protein quantification as described above.

### Bgl2 assay

*SEC3, sec3*Δ*, sec3-mutS* and *sec3*Δ*N* yeast cells were grown at 25 °C to early log phase and shifted for 2 h at 37 °C. After the shift, NaN_3_ and NaF were added to a final concentration of 20 mM. A total of 25 O.D.s of cells were collected, washed with a Tris/NaN3/NaF buffer and spheroplasted. The amount of external and internal Bgl2 was determined by western blotting with an anti-Bgl2 antibody. As a control for loading, the amount of Adh1 from total cell lysates was determined by western blotting. ImageJ software was used for protein quantification as described above. Student's *t*-test was used for statistical analyses.

### Invertase assay

Cells were grown to early log phase in synthetic complete medium overnight at 25 °C. 2.0 OD_600_ units of cells were collected for each sample. Half of the sample was immediately pelleted, resuspended in 1 ml of ice-cold 10 mM NaN_3_ solution, and stored on ice. The other half was incubated in YP (1% yeast extract+2% peptone) medium plus 0.1% glucose for 2 h at 25 °C for invertase induction. Measurement of internal and external invertase activity was performed on all samples as described previously^63^. Student's *t*-test was used for statistical analyses.

### Electronic microscopy

Electronic Microscopy was performed as previously described^32^. In brief, yeast cells were spun down and resuspended in 1 ml of 3% glutaraldehyde, 0.1 M cacodylate containing 5 mM CaCl_2_ and 5 mM MgCl_2_ for 1 h. Cells were dispersed and subsequently embedded in 2% low melting point agarose (1:1), cooled, and cut into small pieces (about 1 mm^3^). Blocks are post-fixed in 4% KMnO_4_ for 1 h and treated with 0.5% sodium metaperiodate for 15 min at room temperature. Blocks were washed twice in ddH_2_O, and placed into filtered 2% uranyl acetate overnight. Blocks were dehydrated through a graded series of ethanol (50–100%) overnight, washed with propylene oxide, and placed into 50:50 mixture of propylene oxide and Spurr resin and then 100% Spurr resin for embedding. Cells were observed using a Model 1010 JEOL transmission microscopy at 100,000 × magnification.

### Fluorescence microscopy

Fluorescence microscopy was performed with a Leica CTR6000 fluorescence microscope equipped with a Plan-Apochromat 100 × , 1.40 NA oil immersion objective lens. Images were taken using LAS AF 1.5.1 acquisition software (Leica). Cells were grown to early log phase (0.6 OD_600_) in synthetic complete media and fixed by methanol/acetone before microscopy. Images were captured with a digital camera (DFC350FX; Leica) at room temperature.

### Ectopic recruitment of secretory vesicles to mitochondria

The ectopic targeting assay was performed as previously described^32^. Briefly, *SEC3* and *sec3mutS* were subcloned into the Tom20-mCherry vector (pGV373) using the SalI/SalI restriction sites. Tom20-mCherry-*SEC3* (pG1872) and Tom20-mCherry-*sec3mutS1* (pG1942) were used to transform yeast strain NY1490. The mitochondria marker Cit1-GFP was co-expressed with Tom20-mCherry-tagged Sec3 variants to confirm their mitochondria localization. Sec6-GFP and Sec8-GFP were generated by chromosomal integration in strains expressing Tom20-mCherry-tagged Sec3 variants, and observed directly in yeast cells. Sec4 was detected by immunostaining with a rabbit polyclonal antibody after yeast cell spheroplasting. Mitochondria clustered in yeast cells expressing Tom20-tagged Sec3 as previously explained^32^. The number of co-localized cells were counted (50 cells, three times) and plotted. Student's *t*-test was used for statistical analyses.

### Data availability

Coordinates and structure factors for the C222_1_ and P4_3_2_1_2 crystal forms of the Sec3N–Sso2 complex have been deposited in the Protein Data Bank with accession numbers 5M4Y and 5LG4, respectively. All other data that support the conclusions of the study are available from the corresponding author.

## Additional information

**How to cite this article:** Yue, P. *et al*. Sec3 promotes the initial binary t-SNARE complex assembly and membrane fusion. *Nat. Commun.* **8,** 14236 doi: 10.1038/ncomms14236 (2017).

**Publisher's note:** Springer Nature remains neutral with regard to jurisdictional claims in published maps and institutional affiliations.

## Supplementary Material

Supplementary Information Supplementary Figures, Supplementary Tables

## References

[b1] Rothman J. E. Mechanisms of intracellular protein transport. Nature 372, 55–63 (1994). 7969419 10.1038/372055a0

[b2] Sutton R. B., Fasshauer D., Jahn R. & Brunger A. T. Crystal structure of a SNARE complex involved in synaptic exocytosis at 2.4A resolution. Nature 395, 347–353 (1998). 9759724 10.1038/26412

[b3] Fernandez I. . Three-dimensional structure of an evolutionarily conserved N-terminal domain of syntaxin 1A. Cell 94, 841–849 (1998). 9753330 10.1016/s0092-8674(00)81742-0

[b4] Nicholson K. L. . Regulation of SNARE complex assembly by an N-terminal domain of the t-SNARE Sso1p. Nat. Struct. Biol. 5, 793–802 (1998). 9731774 10.1038/1834

[b5] Dulubova I. . A conformational switch in syntaxin during exocytosis: role of munc18. EMBO J. 18, 4372–4382 (1999). 10449403 10.1093/emboj/18.16.4372 PMC1171512

[b6] Fiebig K. M., Rice L. M., Pollock E. & Brunger A. T. Folding intermediates of SNARE complex assembly. Nat. Struct. Biol. 6, 117–123 (1999). 10048921 10.1038/5803

[b7] Parlati F. . Rapid and efficient fusion of phospholipid vesicles by the alpha-helical core of a SNARE complex in the absence of an N-terminal regulatory domain. Proc. Natl Acad. Sci. USA 96, 12565–12570 (1999). 10535962 10.1073/pnas.96.22.12565 PMC22992

[b8] Munson M., Chen X., Cocina A. E., Schultz S. M. & Hughson F. M. Interactions within the yeast t-SNARE Sso1p that control SNARE complex assembly. Nat. Struct. Biol. 7, 894–902 (2000). 11017200 10.1038/79659

[b9] Van Komen J. S., Bai X., Scott B. L. & McNew J. A. An intramolecular t-SNARE complex functions in vivo without the syntaxin NH2-terminal regulatory domain. J. Cell Biol. 172, 295–307 (2006). 16401725 10.1083/jcb.200507138 PMC2063558

[b10] Demircioglu F. E., Burkhardt P. & Fasshauer D. The SM protein Sly1 accelerates assembly of the ER-Golgi SNARE complex. Proc. Natl Acad. Sci. USA 111, 13828–13833 (2014). 25189771 10.1073/pnas.1408254111 PMC4183299

[b11] Gerber S. H. . Conformational switch of syntaxin-1 controls synaptic vesicle fusion. Science 321, 1507–1510 (2008). 18703708 10.1126/science.1163174 PMC3235364

[b12] Pfeffer S. R. Transport-vesicle targeting: tethers before SNAREs. Nat. Cell Biol. 1, E17–E22 (1999). 10559876 10.1038/8967

[b13] Whyte J. R. & Munro S. Vesicle tethering complexes in membrane traffic. J. Cell Sci. 115, 2627–2637 (2002). 12077354 10.1242/jcs.115.13.2627

[b14] Yu I. M. & Hughson F. M. Tethering factors as organizers of intracellular vesicular traffic. Annu. Rev. Cell Dev. Biol. 26, 137–156 (2010). 19575650 10.1146/annurev.cellbio.042308.113327

[b15] Munson M. & Novick P. The exocyst defrocked, a framework of rods revealed. Nat. Struct. Mol. Biol. 13, 577–581 (2006). 16826234 10.1038/nsmb1097

[b16] He B. & Guo W. The exocyst complex in polarized exocytosis. Curr. Opin. Cell Biol. 21, 537–542 (2009). 19473826 10.1016/j.ceb.2009.04.007 PMC2725219

[b17] Wu B. & Guo W. The exocyst at a glance. J. Cell Sci. 128, 2957–2964 (2015). 26240175 10.1242/jcs.156398 PMC4541039

[b18] Novick P., Field C. & Schekman R. Identification of 23 complementation groups required for post-translational events in the yeast secretory pathway. Cell 21, 205–215 (1980). 6996832 10.1016/0092-8674(80)90128-2

[b19] TerBush D. R. & Novick P. Sec6, Sec8, and Sec15 are components of a multisubunit complex which localizes to small bud tips in Saccharomyces cerevisiae. J. Cell Biol. 130, 299–312 (1995). 7615633 10.1083/jcb.130.2.299 PMC2199927

[b20] Brennwald P. . Sec9 is a SNAP-25-like component of a yeast SNARE complex that may be the effector of Sec4 function in exocytosis. Cell 79, 245–258 (1994). 7954793 10.1016/0092-8674(94)90194-5

[b21] Scott B. L. . Liposome fusion assay to monitor intracellular membrane fusion machines. Methods Enzymol. 372, 274–300 (2003). 14610819 10.1016/S0076-6879(03)72016-3

[b22] Yu H. . Comparative studies of Munc18c and Munc18-1 reveal conserved and divergent mechanisms of Sec1/Munc18 proteins. Proc. Natl Acad. Sci. USA 110, E3271–E3280 (2013). 23918365 10.1073/pnas.1311232110 PMC3761595

[b23] Baek K. . Structure-function study of the N-terminal domain of exocyst subunit Sec3. J. Biol. Chem. 285, 10424–10433 (2010). 20139078 10.1074/jbc.M109.096966 PMC2856249

[b24] Yamashita M. . Structural basis for the Rho- and phosphoinositide-dependent localization of the exocyst subunit Sec3. Nat. Struc. Mol. Biol. 17, 180–186 (2010). 10.1038/nsmb.1722 20062059

[b25] Misura K. M., Scheller R. H. & Weis W. I. Three-dimensional structure of the neuronal-Sec1-syntaxin 1a complex. Nature 404, 355–362 (2000). 10746715 10.1038/35006120

[b26] Strop P., Kaiser S. E., Vrljic M. & Brunger A. T. The structure of the yeast plasma membrane SNARE complex reveals destabilizing water-filled cavities. J. Biol. Chem. 283, 1113–1119 (2008). 17956869 10.1074/jbc.M707912200

[b27] Boyd C., Hughes T., Pypaert M. & Novick P. Vesicles carry most exocyst subunits to exocytic sites marked by the remaining two subunits, Sec3p and Exo70p. J. Cell Biol. 167, 889–901 (2004). 15583031 10.1083/jcb.200408124 PMC2172445

[b28] Zhang X. . Membrane association and functional regulation of Sec3 by phospholipids and Cdc42. J. Cell Biol. 180, 145–158 (2008). 18195105 10.1083/jcb.200704128 PMC2213614

[b29] Guo W., Tamanoi F. & Novick P. Spatial regulation of the exocyst complex by Rho1 GTPase. Nat. Cell Biol. 3, 353–360 (2001). 11283608 10.1038/35070029

[b30] Wiederkehr A., Du Y., Pypaert M., Ferro-Novick S. & Novick P. Sec3p is needed for the spatial regulation of secretion and for the inheritance of the cortical endoplasmic reticulum. Mol. Biol. Cell 14, 4770–4782 (2003). 12960429 10.1091/mbc.E03-04-0229 PMC284782

[b31] Willett R. . COG complexes form spatial landmarks for distinct SNARE complexes. Nat. Commun. 4, 1553 (2013). 23462996 10.1038/ncomms2535 PMC3595136

[b32] Luo G., Zhang J. & Guo W. The role of Sec3p in secretory vesicle targeting and exocyst complex assembly. Mol. Biol. Cell 25, 3813–3822 (2014). 25232005 10.1091/mbc.E14-04-0907 PMC4230786

[b33] Wong M. & Munro S. Membrane trafficking. The specificity of vesicle traffic to the Golgi is encoded in the golgin coiled-coil proteins. Science 346, 1256898 (2014). 25359980 10.1126/science.1256898 PMC4254398

[b34] Südhof T. C. & Rizo J. Synaptic vesicle exocytosis. Cold Spring Harb. Perspect. Biol 3, a005637 (2011). 22026965 10.1101/cshperspect.a005637 PMC3225952

[b35] Südhof T. C. Neurotransmitter release: the last millisecond in the life of a synaptic vesicle. Neuron 80, 675–690 (2013). 24183019 10.1016/j.neuron.2013.10.022 PMC3866025

[b36] Lang T. . SNAREs are concentrated in cholesterol-dependent clusters that define docking and fusion sites for exocytosis. EMBO J. 20, 2202–2213 (2001). 11331586 10.1093/emboj/20.9.2202 PMC125434

[b37] Bar-On D. . Super-resolution imaging reveals the internal architecture of nano-sized syntaxin clusters. J. Biol. Chem. 287, 27158–27167 (2012). 22700970 10.1074/jbc.M112.353250 PMC3411058

[b38] Munson M. & Hughson F. M. Conformational regulation of SNARE assembly and disassembly in vivo. J. Biol. Chem. 277, 9375–9381 (2002). 11777922 10.1074/jbc.M111729200

[b39] Ren Y. . A structure-based mechanism for vesicle capture by the multisubunit tethering complex Dsl1. Cell 139, 1119–1129 (2009). 20005805 10.1016/j.cell.2009.11.002 PMC2806190

[b40] Tripathi A., Ren Y., Jeffrey P. D. & Hughson F. M. Structural characterization of Tip20p and Dsl1p, subunits of the Dsl1p vesicle tethering complex. Nat. Struct. Mol. Biol. 16, 114–123 (2009). 19151722 10.1038/nsmb.1548 PMC2635920

[b41] Stroupe C., Collins K. M., Fratti R. A. & Wickner W. Purification of active HOPS complex reveals its affinities for phosphoinositides and the SNARE Vam7p. EMBO J. 25, 1579–1589 (2006). 16601699 10.1038/sj.emboj.7601051 PMC1440844

[b42] Zick M. & Wickner W. The tethering complex HOPS catalyzes assembly of the soluble SNARE Vam7 into fusogenic trans-SNARE complexes. Mol. Biol. Cell 24, 3746–3753 (2013). 24088569 10.1091/mbc.E13-07-0419 PMC3843000

[b43] Sivaram M. V., Saporita J. A., Furgason M. L., Boettcher A. J. & Munson M. Dimerization of the exocyst protein Sec6p and its interaction with the t-SNARE Sec9p. Biochemistry 44, 6302–6311 (2005). 15835919 10.1021/bi048008z

[b44] Shen D. . The synaptobrevin homologue Snc2p recruits the exocyst to secretory vesicles by binding to Sec6p. J. Cell Biol. 202, 509–526 (2013). 23897890 10.1083/jcb.201211148 PMC3734085

[b45] Dubuke M. L., Maniatis S., Shaffer S. A. & Munson M. The exocyst subunit Sec6 interacts with assembled exocytic SNARE complexes. J. Biol. Chem. 115, 673806 (2015). 10.1074/jbc.M115.673806 PMC4653681 26446795

[b46] Shorter J., Beard M. B., Seemann J., Dirac-Svejstrup A. B. & Warren G. Sequential tethering of Golgins and catalysis of SNAREpin assembly by the vesicle-tethering protein p115. J. Cell Biol. 157, 45–62 (2002). 11927603 10.1083/jcb.200112127 PMC2173270

[b47] Puthenveedu M. A. & Linstedt A. D. Gene replacement reveals that p115/SNARE interactions are essential for Golgi biogenesis. Proc. Natl Acad. Sci. USA 101, 1253–1256 (2004). 14736916 10.1073/pnas.0306373101 PMC337039

[b48] Wang T., Grabski R., Sztul E. & Hay J. C. p115-SNARE interactions: a dynamic cycle of p115 binding monomeric SNARE motifs and releasing assembled bundles. Traffic 16, 148–171 (2015). 25406594 10.1111/tra.12242 PMC4304910

[b49] Südhof T. C. & Rothman J. E. Membrane fusion: grappling with SNARE and SM proteins. Science 323, 474–477 (2009). 19164740 10.1126/science.1161748 PMC3736821

[b50] Rizo J. & Südhof T. C. The membrane fusion enigma: SNAREs, Sec1/Munc18 proteins, and their accomplices--guilty as charged? Annu. Rev. Cell Dev. Biol. 28, 279–308 (2012). 23057743 10.1146/annurev-cellbio-101011-155818

[b51] Baker R. W. . A direct role for the Sec1/Munc18-family protein Vps33 as a template for SNARE assembly. Science. 349, 1111–1114 (2015). 26339030 10.1126/science.aac7906 PMC4727825

[b52] Carr C. M., Grote E., Munson M., Hughson F. M. & Novick P. J. Sec1p binds to SNARE complexes and concentrates at sites of secretion. J. Cell Biol. 146, 333–344 (1999). 10427089 10.1083/jcb.146.2.333 PMC3206579

[b53] Hashizume K., Cheng Y. S., Hutton J. L., Chiu C. H. & Carr C. M. Yeast Sec1p functions before and after vesicle docking. Mol. Biol. Cell 20, 4673–4685 (2009). 19776355 10.1091/mbc.E09-02-0172 PMC2777098

[b54] Carr C. M. & Rizo J. At the junction of SNARE and SM protein function. Curr. Opin. Cell Biol. 22, 488–495 (2010). 20471239 10.1016/j.ceb.2010.04.006 PMC2923694

[b55] Gallwitz D. & Jahn R. The riddle of the Sec1/Munc-18 proteins-new twists added to their interactions with SNAREs. Trends Biochem. Sci. 28, 113–116 (2003). 12633987 10.1016/S0968-0004(03)00028-8

[b56] Wiederkehr A., De Craene J. O., Ferro-Novick S. & Novick P. J. Functional specialization within a vesicle tethering complex: bypass of a subset of exocyst deletion mutants by Sec1p or Sec4p. J. Cell Biol. 167, 875–887 (2004). 15583030 10.1083/jcb.200408001 PMC2172455

[b57] Morgera F. . Regulation of exocytosis by the exocyst subunit Sec6 and the SM protein Sec1. Mol. Biol. Cell 23, 337–346 (2012). 22114349 10.1091/mbc.E11-08-0670 PMC3258177

[b58] Li W. . The crystal structure of a Munc13 C-terminal module exhibits a remarkable similarity to vesicle tethering factors. Structure 19, 1443–1455 (2011). 22000513 10.1016/j.str.2011.07.012 PMC3197213

[b59] Ma C., Su L., Seven A. B., Xu Y. & Rizo J. Reconstitution of the vital functions of Munc18 and Munc13 in neurotransmitter release. Science 339, 421–425 (2013). 23258414 10.1126/science.1230473 PMC3733786

[b60] Yang X. . Syntaxin opening by the MUN domain underlies the function of Munc13 in synaptic-vesicle priming. Nat. Struct. Mol. Biol. 22, 547–554 (2015). 26030875 10.1038/nsmb.3038 PMC4809529

[b61] Munson M. Show me the MUN-y. Structure 19, 1348–1349 (2011). 22000505 10.1016/j.str.2011.09.004 PMC3202169

[b62] Pei J., Ma C., Rizo J. & Grishin N. V. Remote homology between Munc13 MUN domain and vesicle tethering complexes. J. Mol. Biol. 391, 509–517 (2009). 19563813 10.1016/j.jmb.2009.06.054 PMC3158588

[b63] He B., Xi F., Zhang X., Zhang J. & Guo W. Exo70 interacts with phospholipids and mediates the targeting of the exocyst to the plasma membrane. EMBO J. 26, 4053–4065 (2007). 17717527 10.1038/sj.emboj.7601834 PMC2230670

[b64] Liu D. & Novick P. J. Bem1p contributes to secretory pathway polarization through a direct interaction with Exo70p. J. Cell Biol. 207, 59–72 (2014). 25313406 10.1083/jcb.201404122 PMC4195821

[b65] Finger F. P., Hughes T. E. & Novick P. Sec3p is a spatial landmark for polarized secretion in budding yeast. Cell 92, 559–571 (1998). 9491896 10.1016/s0092-8674(00)80948-4

[b66] Kabsch W. XDS. Acta Crystallogr. D, Biol. Crystallogr. 66, 125–132 (2010). 20124692 10.1107/S0907444909047337 PMC2815665

[b67] McCoy A. J. . Phaser crystallographic software. J. Appl. Crystallogr. 40, 658–674 (2007). 19461840 10.1107/S0021889807021206 PMC2483472

[b68] Emsley P. & Cowtan K. Coot: model-building tools for molecular graphics. Acta Crystallogr. D, Biol. Crystallogr. 60, 2126–2132 (2004). 15572765 10.1107/S0907444904019158

[b69] Terwilliger T. C. . Decision-making in structure solution using Bayesian estimates of map quality: the PHENIX AutoSol wizard. Acta Crystallogr. D, Biol. Crystallogr. 65, 582–601 (2009). 19465773 10.1107/S0907444909012098 PMC2685735

[b70] Ericsson U. B., Hallberg B. M., Detitta G. T., Dekker N. & Nordlund P. Thermofluor-based high-throughput stability optimization of proteins for structural studies. Anal. Biochem. 357, 289–298 (2006). 16962548 10.1016/j.ab.2006.07.027

