# Supplementary material for: Sec3 promotes the initial binary t-SNARE complex assembly and membrane fusion
Source: Nat Commun. 2017 Jan 23;8:14236. doi: 10.1038/ncomms14236 (PMC5267525; doi:10.1038/ncomms14236)
Supplement: Supplementary Information — Supplementary Figures, Supplementary Tables [file ncomms14236-s1.pdf]

SUPPLEMENTARY INFORMATION

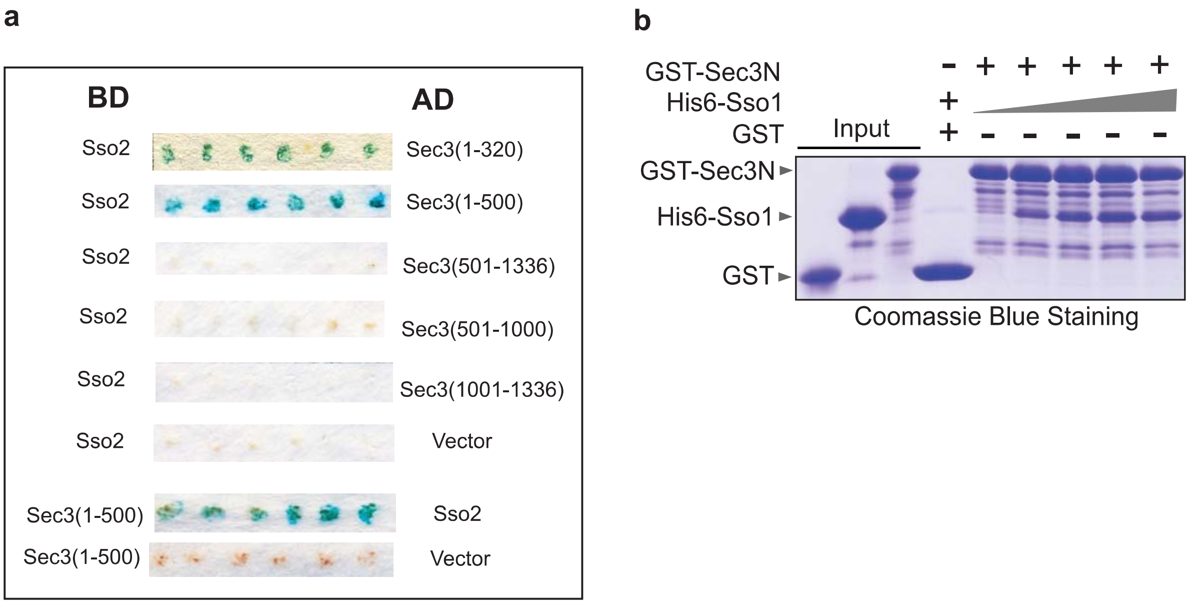

Supplementary Figure 1. (a) Yeast 2-hybrid assays show the interaction between Sec3 and Sso2. Sso2 and different domains of Sec3 were cloned into pAS1-CYH (yeast 2-hybrid DNA-binding domain vector) or pACT2 (yeast 2-hybrid activation domain vector). Yeast strain Y190 was co-transformed with Sso2 and Sec3 plasmids and grown on synthetic complete plates lacking leucine and histidine. X-Gal assay was performed to reveal the interaction. **(b)** Purified recombinant Sso1 (2, 4, 8, 16 μM) was incubated with 60 μg of GST-Sec3N conjugated to glutathione Sepharose 4B in a reaction volume of 200 μl. Aliquots of the bound reactions were analyzed by SDS-PAGE and Coomassie Blue staining.

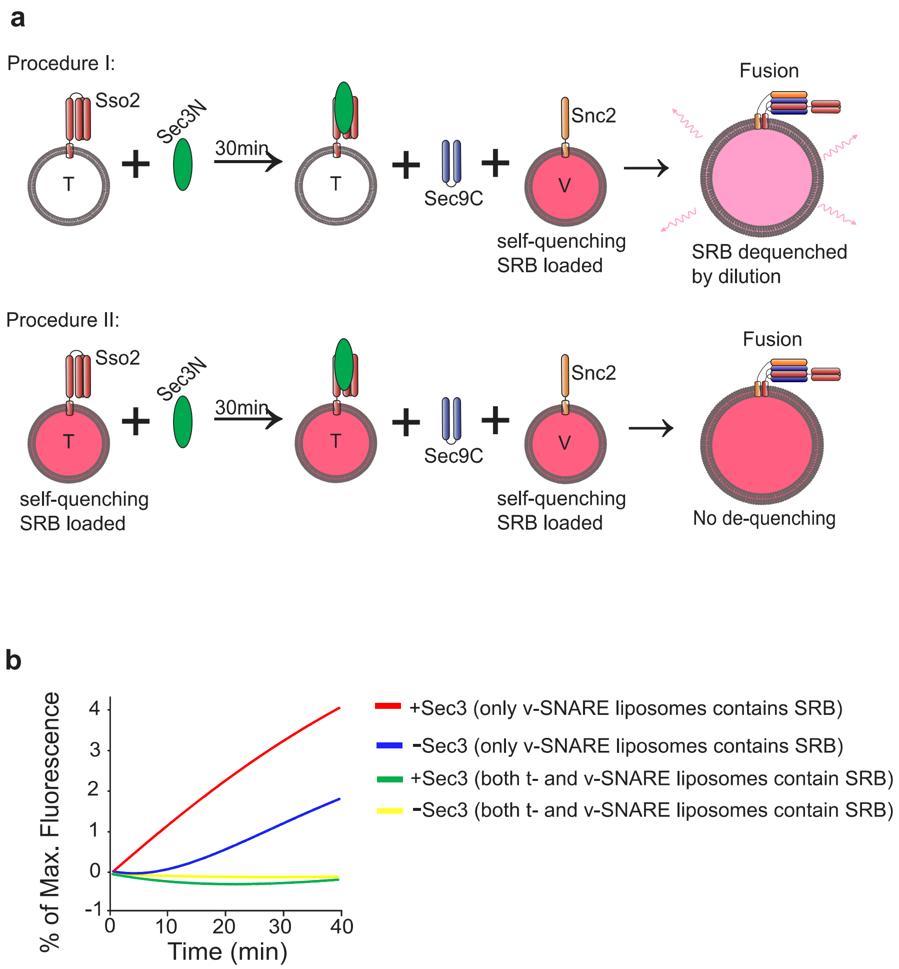

Supplementary Figure 2. Sec3N promotes liposome fusion without causing leakage. **(a)** Schematic diagram showing the procedures used in the fusion experiments. Procedure I: The t-SNARE liposomes were reconstituted with Sso2. The v-SNARE liposomes were reconstituted with Snc2 and encapsulated soluble sulforhodamine B (SRB) at a self-quenching concentration of 50 mM. The t-SNARE liposomes were pre-incubated with GST-Sec3N (5μM) or GST (5μM) at 25°C for 30 min, and then incubated with His6-Sec9C (5μM) and SRB-containing v-SNARE loposomes at 37°C to start fusion. If fusion occurs, the dilution of SRB in the fused vesicles will lead to emission of fluorescence due to de-quenching. Procedure II: To test whether liposomes leaked during the reaction, both the t-SNARE and v-SNARE liposomes were loaded with SRB at a self-quenching concentration (50 mM). Throughout the fusion reaction, SRB will not become diluted, thus there should not be emission of fluorescence due to de-quenching. **(b)** Sec3N showed stimulatory effect on liposome fusion (red *vs*. blue curves) when following Procedure I. No leakage was detected in the fusion reaction (green *vs*. yellow curves) when following Procedure II. The data were plotted by fitting to single rectangular hyperbola equation using SigmaPlot. The result represents 3 assays.

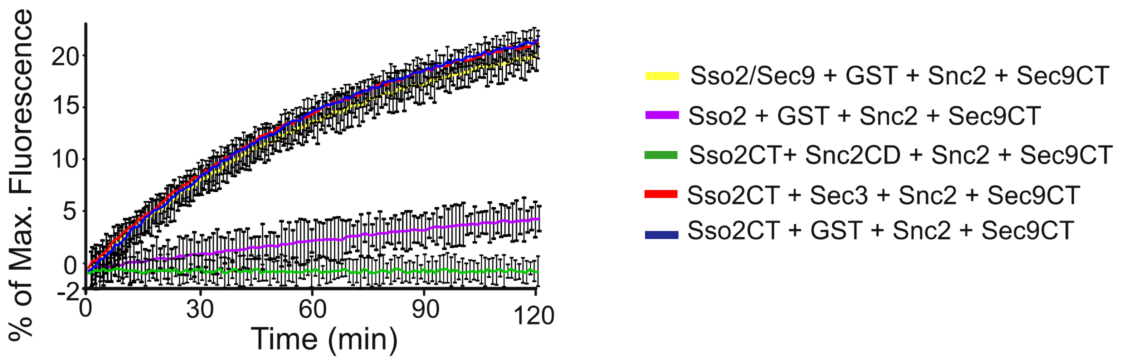

Supplementary Figure 3. Liposomes with Sso2CT have a faster fusion rate. The t-SNARE liposomes were reconstituted with Sso2 (yellow and purple) or Sso2CT (green, red, and blue). The v-SNARE liposomes were reconstituted with Snc2. The fusion rate with Sso2CT liposomes (blue) was comparable to the full-length Sso2 liposomes pre-assembled with Sec9CT (yellow), and was faster than Sso2 reaction without pre-assembled Sec9 (purple). $P<0.01$ at 30 min, 60 min, 90 min time points. Sec3N has no further stimulatory effect on Sso2CT-mediated fusion (red). Addition of Snc2CD inhibited the fusion reaction (green line). The graphs represent the averages of three experiments. Error bars, standard deviation.

a

Hydrogen bonds

Sec3		Sso2
(K149-NZ	..H_2O..	R134-NE)
Q219-OE1	......	N219-ND2
E222-O	......	S138-OG
E222-OE1	......	K141-NZ
H224-NE2	......	E142-OE2
F236-O	......	Q131-NE2
Y237-OH	......	E222-OE1
Y237-OH	......	E222-OE2
R241-NH1	......	N59-OD1
R245-NH1	......	D62-OD2
R245-NH2	......	D62-OD1
R245-O	......	N139-ND2
R245-NE	......	N139-OD1

Salt bridges

Sec3		Sso2
E222-OE1	+++	K141-NZ
E222-OE2	+++	K141-NZ
H224-NE2	+++	E142-OE1
H224-NE2	+++	E142-OE2
D239-OD1	+++	K128-NZ
D239-OD2	+++	K128-NZ
R245-NH1	+++	D62-OD1
R245-NH1	+++	D62-OD2
R245-NH2	+++	D62-OD1
R245-NH2	+++	D62-OD2

b

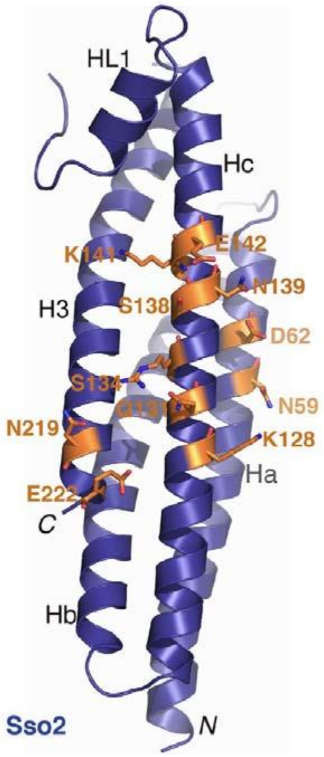

Supplementary Figure 4. (a) List of hydrogen bonds and salt bridges across the Sec3/Sso2 binding interface in the crystal structure. Note that the interaction between residue K149 of Sec3 and R134 of Sso2 is through water-mediated hydrogen bonds. (**b**) Residues on Sso2 interacting with Sec3 were highlighted.

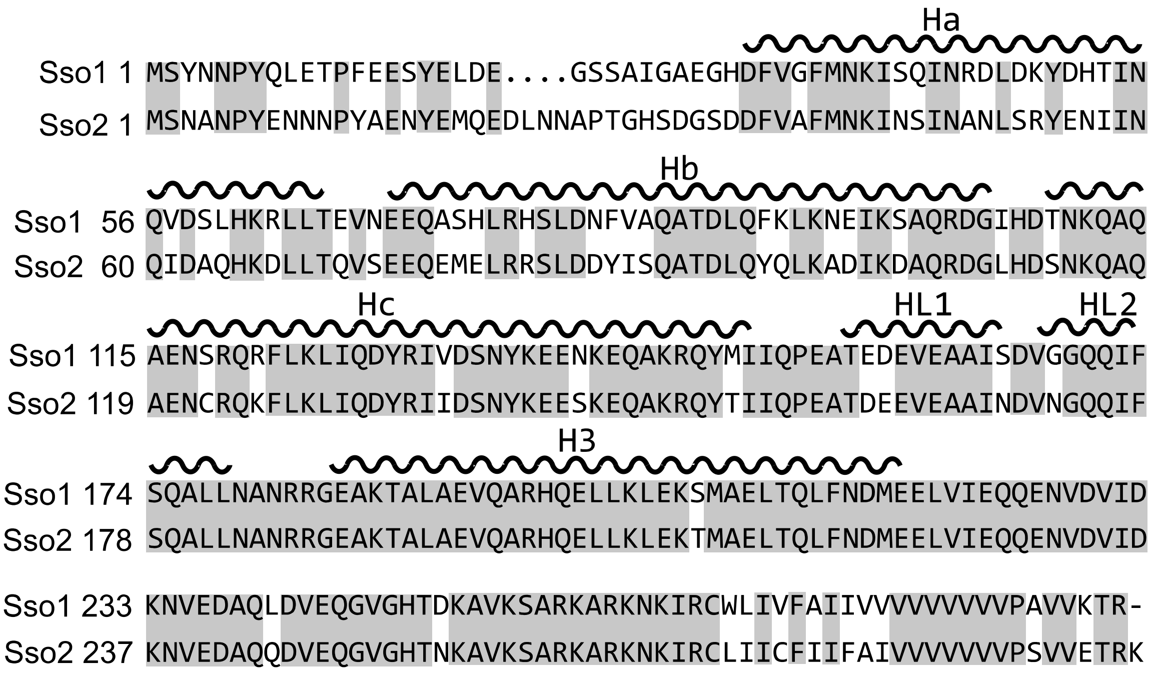

Supplementary Figure 5. Sequence alignment of Sso1 and Sso2. Secondary structures are shown above the sequences.

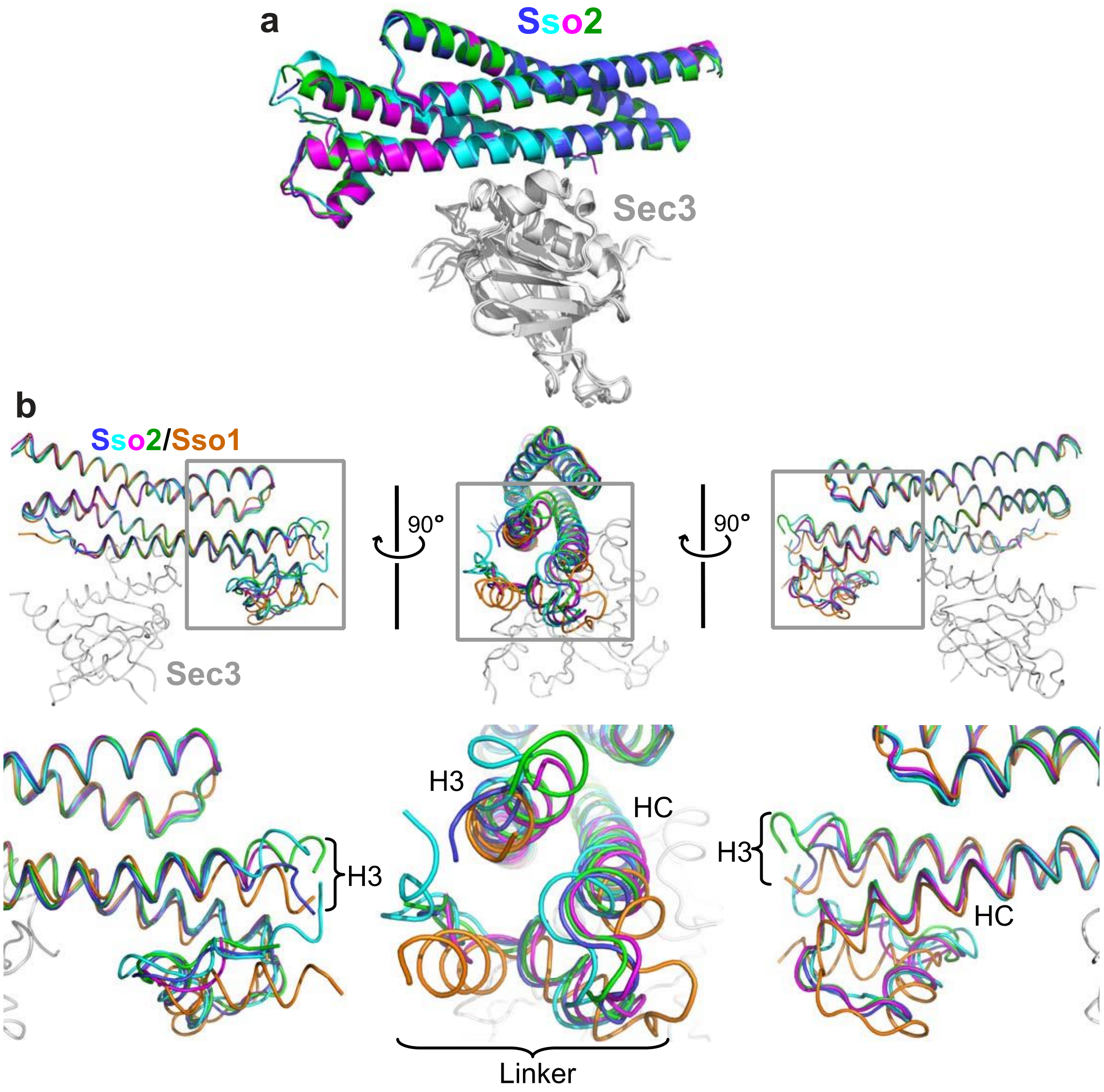

Supplementary Figure 6. Comparison of the two crystal forms of the Sec3/Sso2 complex. (**a**) Overlay of the four copies of the Sec3/Sso2 complex present in the two different crystal forms, with Sso2 molecules superimposed onto each another. The overlay shows that the binding interfaces in the four dimers are essentially the same. (**b**) Structural comparison of Sso1 (1FIO.pdb) and the four copies of Sso2 present in the two different crystal forms of the Sso2/Sec3 complex. Three orthogonal views are displayed to show the overall structural changes in the linker region. Boxed regions are enlarged under the images for clarity. The junctions in all Sec3-bound Sso2s show a similar conformation, which is distinct from that of Sso1 alone. The structures are shown in the same color scheme as in (**a**).

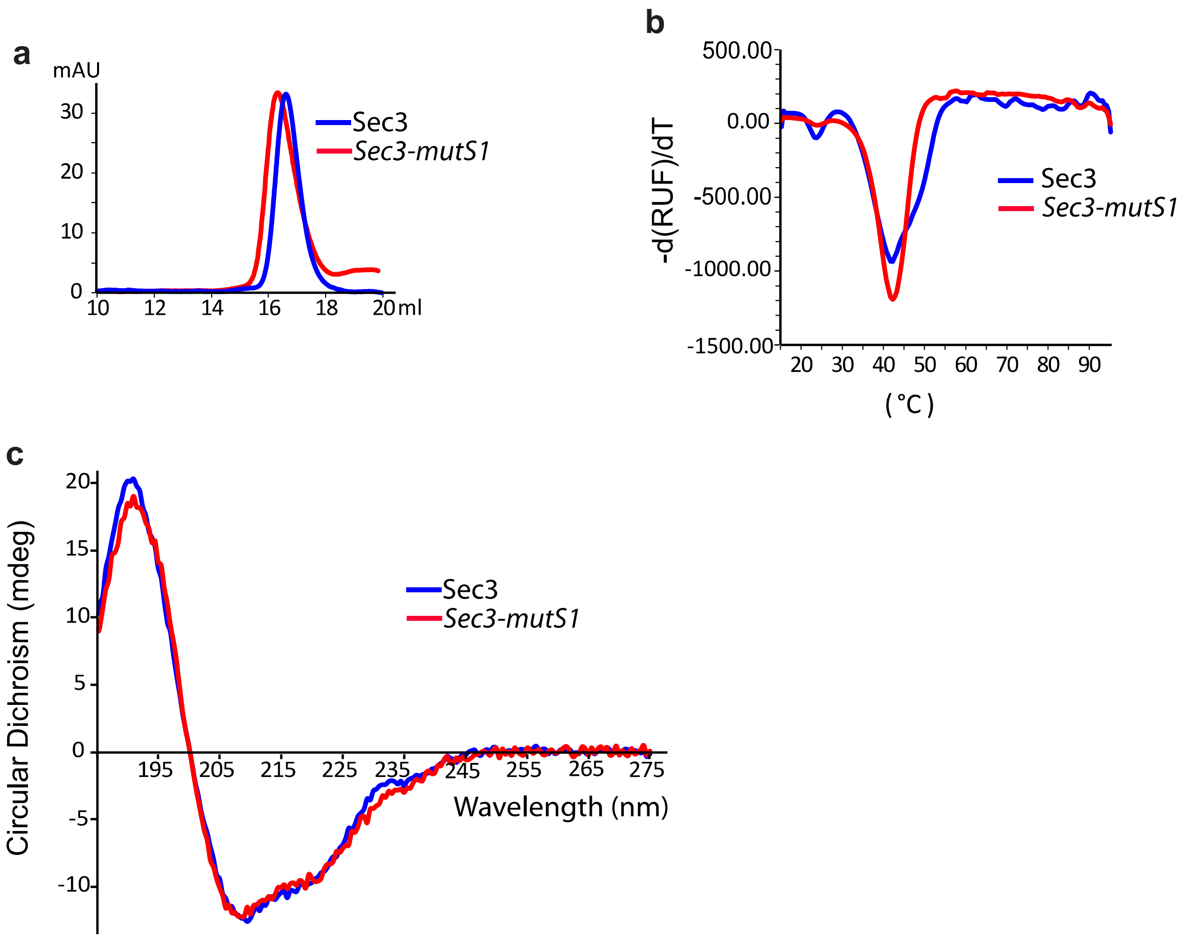

Supplementary Figure 7. Mutagenesis in *Sec3-mutS1* do not cause overall structure change of Sec3N. **(a)** Elution profiles of Sec3 and *Sec3-mutS1* from an analytical SEC column (Superdex-200 10/300 GL). **(b)** Melting curves of Sec3 and *Sec3-mutS1* measured by Thermofluor assays. The melting temperatures for both proteins are 42.0 °C. **(c)** Far-UV CD spectra of purified Sec3 and *Sec3-mutS1* show essentially the same folding for both proteins.

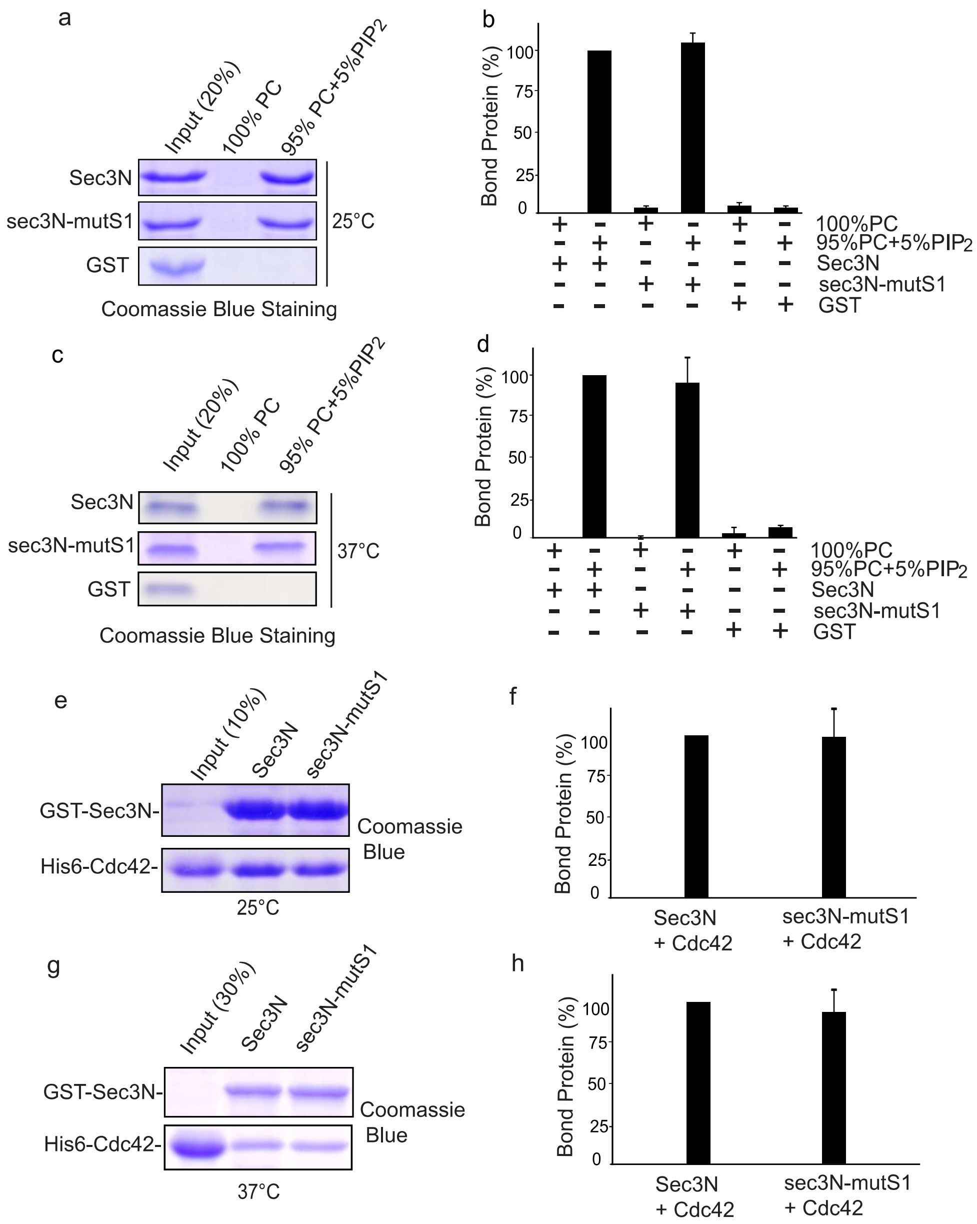

Supplementary Figure 8. Disruption of the Sec3-Sso2 interaction does not affect Sec3-$PI(4,5)P_2$ interaction and Sec3-Cdc42 interaction. GST, Sec3N, and Sec3N-mutS1 were incubated with large unilamellar vesicles (LUV) containing 95% PC and 5% $PI(4,5)P_2$ at 25°C (**a** and **b**) or 37°C (**c** and **d**). Proteins associated with liposomes were resolved by SDS-PAGE and detected by Coomassie Blue staining. Quantification of the binding, which was normalized to wild type Sec3N binding, is shown in (**b**) and (**d**) (n=3; $P>0.1$ between Sec3N and Sec3N-mutS1). GST-tagged wild type and mutant Sec3N were incubated with His6-Cdc42 for binding assay at 25°C (**e** and **f**) or 37°C (**g** and **h**). Cdc42 bound to Sec3 were resolved by SDS-PAGE and detected by Coomassie Blue staining. Quantification of the binding, which was normalized to wild type Sec3N binding, is shown in (**f**) and (**h**) (n=3; $P>0.1$ between Sec3N and Sec3N-mutS1).

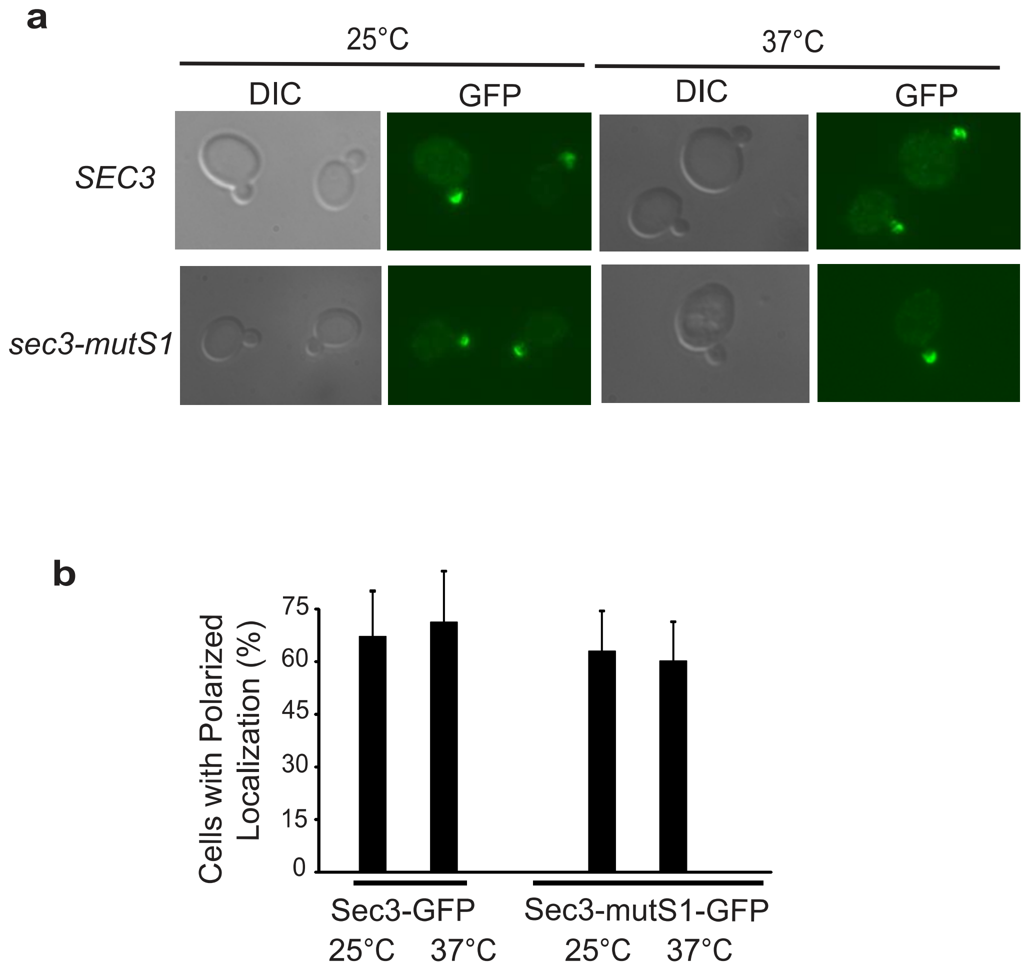

Supplementary Figure 9. (**a**) Disruption of the Sec3-Sso2 interaction does not affect polarized localization of Sec3 in yeast cells. *SEC3* and *sec3-mutS1* were tagged by GFP and expressed under *SEC3* promoter in the *sec3Δ* strain. The Sec3-GFP and *sec3-mutS1*-GFP in yeast cells were examined using fluorescence microscopy. (**b**) Quantification of the percentage of cells with polarized localization of GFP-Sec3 and GFP-*sec3-mutS1* at the bud tip or mother-daughter cell junctions. Student's t-test was used for statistical analyses. Three independent experiments were performed. 50 cells were counted in each experiment. P>0.1 comparing the percentage of cells with polarized Sec3-GFP or *sec3-mutS1*-GFP localization.

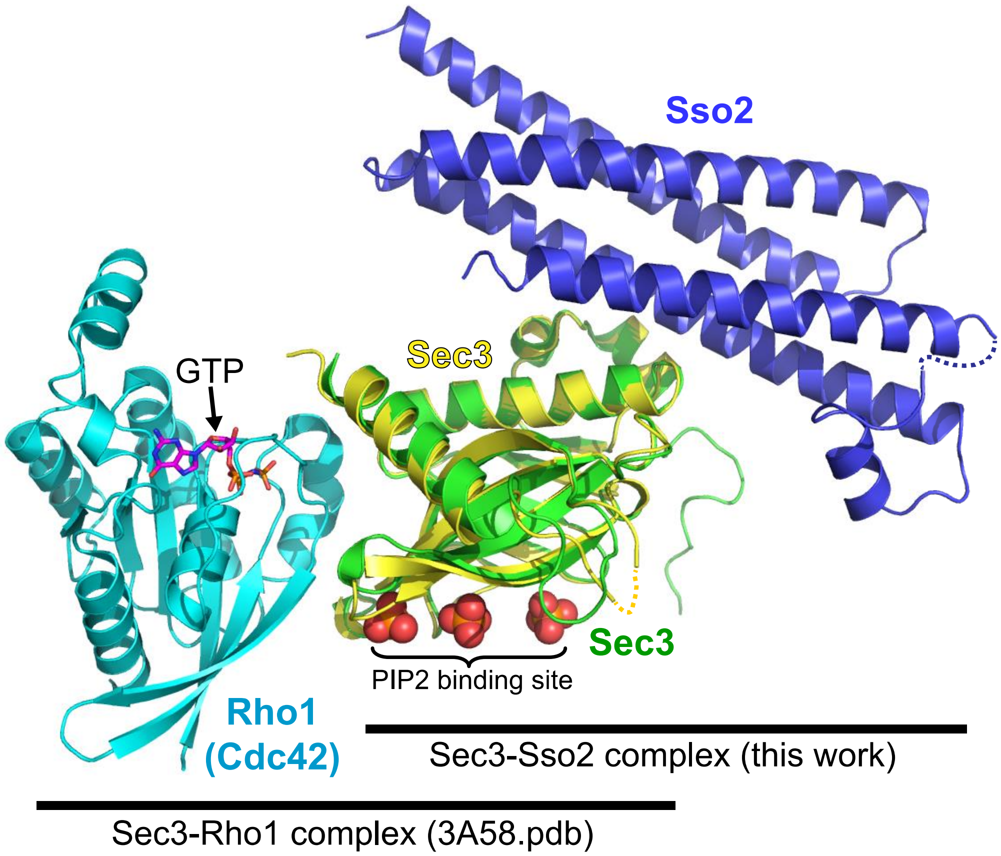

Supplementary Figure 10. Sso2, Rho1 and PI(4,5)P_2 bind to different sites on Sec3N. Crystal structures of Sec3-Sso2 (yellow and blue) and Sec3-Rho1 (green and cyan, PDB code: 3A58) were aligned by superimposing Sec3 molecules. The PH fold of Sec3N from the two structures aligns very well. Sso2 binds to the C-terminal extension of the PH domain that resides at the close end of the β-barrel; Rho1, which share similar structure with Cdc42, binds to one side of the β-barrel and mainly interacts with residues at one end of the β-sheets; PI(4,5)P_2 binds to a highly positive charged region at the open end of the β-barrel. GTP is shown as magenta sticks; phosphate representing PI(4,5)P_2 binding site are indicated as red balls.

a

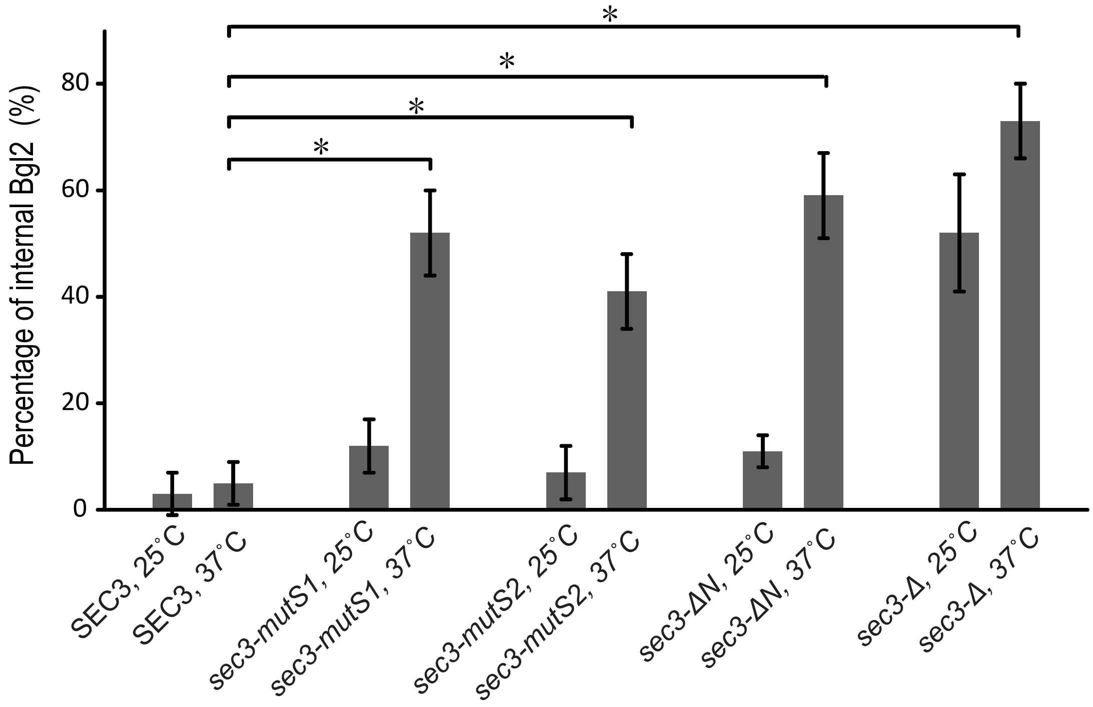

b

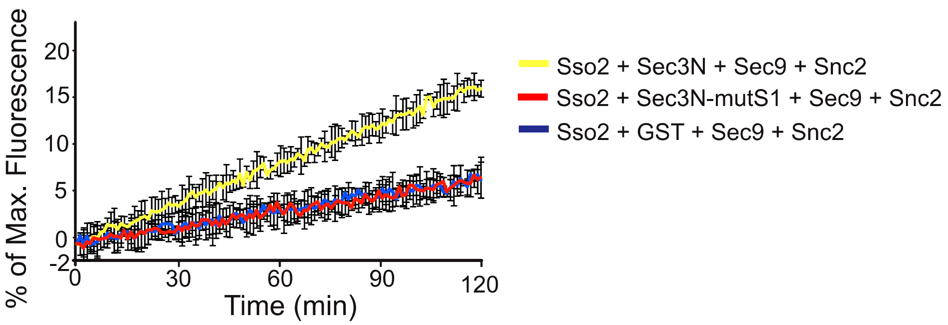

Supplementary Figure 11. (**a**) Quantification of Bgl2 assay shown in Figure 7c. Student's t-test was used for statistical analyses (n=3). "*", P<0.01. (**b**) The t-SNARE liposomes reconstituted with the wild type Sso2 were pre-incubated with Sec3N, Sec3N-mutS1, or GST for 30 min. Sec9CT and the v-SNARE liposomes incorporated with Snc2 were then added for the fusion reaction. The graph represents the average of three experiments. Student's t-test was used for statistical analyses. Error bars, standard deviation. P<0.01 comparing the fusion with Sec3N and Sec3N-mutS1 at 30 min, 60 min, and 90 min time points.

Supplementary Table 1. Data collection and refinement statistics (Molecular Replacement)

	**Crystal form 1**	**Crystal form 2**
**Data collection**		
Space group	$C222_1$	$P4_32_12$
Cell dimensions		
a, *b*, *c* (Å)	101.15, 135.80, 185.74	84.12, 84.12, 237.00
α, β, γ (°)	90, 90, 90	90, 90, 90
Resolution (Å)	50-2.20 (2.33-2.20)*	50-2.90 (3.08-2.90)*
R_{meas}	0.196(1.60)	0.158(2.64)
$I / \sigma I$	13.2 (1.7)	15.3 (1.2)
Completeness (%)	99.9 (99.4)	99.9 (99.4)
Redundancy	13.5 (13.1)	25.6(24.8)
**Refinement**		
Resolution (Å)	20-2.20	20-2.90
No. reflections	64,891	19,614
R_{work} / R_{free}	0.229/0.264	0.263/0.281
No. atoms		
Protein	8,674	4,399
Ligand/ion	6	20
Water	325	-
B-factors (Å^2)		
Protein	48.17	109.25
Ligand/ion	64.70	106.81
Water	42.92	-
R.m.s. deviations		
Bond lengths (Å)	0.002	0.002
Bond angles (°)	0.422	0.523

* Highest resolution shell is shown in parenthesis.

Supplementary Table 2. Plasmids used in this study

Name	Description
pG276	Exo70 a.a.322-623 in pGEX4T-1
pG658	Sec3 a.a. 1-320 in pET-15b
pG707	Sso2 a.a.1-270 in pET15b
pG1067	GST-Sec9CT (a.a.414-651)
pG1215	*sec3*(a.a.307-1336) in *CEN* plasmid, *leu2*$^{-}$
pG1273	*SEC3* in *CEN* plasmid, *leu2*$^{-}$
pG1498	His8-Snc2 in pET15b
pG1509	Sec9CT (a.a.414-651) in pET15b
pG1714	Sso2 in pGEX4T-1
pG1835	Pep12 (a.a.1-263) in pET21C
pG1860	Sec3 (a.a.1-320) in pGEX4T-1
pG1872	p415TEF-TOM20-mCherry-SEC3
pG1915	GST-Sec3N with mutations (K149E, Q219K, E222K, H224D, Y237D, R241E, R245E) ("*sec3-mutS1*")
pG1926	*sec3-mutS1* in *CEN* plasmid, *leu2*$^{-}$
pG1937	*sec3-mutS1*-GFP in *CEN* plasmid, *ura3*$^{-}$
pG1942	p415TEF-TOM20-mCherry-*sec3-mutS1*
pG1945	GST-Sec3N with mutations (K149E, E222K, H224D, Y237D) ("*sec3-mutS2*")
pG1946	*sec3-mutS2* in *CEN* plasmid, *leu2*$^{-}$
pG1971	Sso2-mutHL2 in pET15b
pG1973	Sso2 in p413 GPD vector
pG1974	Sso2-mutHL2 in p413 GPD vector
PNB810	Sec3-GFP in *CEN* plasmid, *ura3*$^{-}$
pNB882	pRS306-SEC6-GFP,digest with BsaBI for chromosomal integration
pNB885	pRS308-SEC6-GFP,digest with BglII for chromosomal integration
pGV373	p415TEF-TOM20-mCherry
pRCLG	pRS416-CIT1-GFP
Sso2-22-15b	Sso2 (a.a.36-227) in pET15b
Sec3-23-MP	MBP-His10-Sec3 a.a.75-320 in MalpET (custom vector)

Supplementary Table 3. Yeast strains used in this study

Name	Genotype
**GY1215**	*ura3-52, leu2-3, 112, his3D200, trp1 Gal^{+}, LA^{+}*
**GY3931**	*ura3-52, leu2-3, 112, his3D200, trp1 Gal^{+}, LA^{+}, sec3Δ::kanMX*
**Y190**	Mat a, *ura3-52, his3-D200, lys2-801, ade2-101, trp1-901, leu2-3*
**NY1490**	Mat a, *ura3-52, leu2-3, 112, his3D200, trp1, Gal^{-}, LA^{-}*
**GY3942**	*Mat a, ura3-52, leu2-3, 112, his3D200, trp1, Gal^{-}, LA^{-}, SEC6-GFP::URA3*
**GY3943**	*Mat a, ura3-52, leu2-3, 112, his3D200, trp1, Gal^{-}, LA^{-}, SEC8-GFP::URA3*
**FHY102**	*Mata his3Δ1 leu2Δ0 ura3Δ0 sso1Δ::kanMX4 sso2Δ::kanMX4 pMM250 (CEN LEU2 SSO1)*
